# Biological sex, sex steroids and sex chromosomes contribute to mouse cardiac aging

**DOI:** 10.18632/aging.205822

**Published:** 2024-05-13

**Authors:** Audrey Morin-Grandmont, Élisabeth Walsh-Wilkinson, Emylie-Ann Labbé, Sara-Ève Thibodeau, Élizabeth Dupont, Dominique K. Boudreau, Marie Arsenault, Yohan Bossé, Jacques Couet

**Affiliations:** 1 Groupe de Recherche sur les Valvulopathies, Centre de Recherche de l’Institut Universitaire de Cardiologie et de Pneumologie de Québec, Université Laval, Québec, Canada

**Keywords:** hypertension, aging, heart failure, mouse, cardiac hypertrophy, sex differences

## Abstract

After menopause, the incidence of cardiovascular disease rapidly rises in women. The disappearing protection provided by sex steroids is a consequence of the development of many risk factors. Preclinical studies are necessary to understand better the effects of ovarian hormones loss cardiac aging.

To mimic menopause in mice and study its consequences, we delayed ovariectomy at 12 months and followed animals for 12 months. Using RNA sequencing, we investigated changes in the myocardial exome with aging. In addition, with four-core genotypes (FCG) transgenic mice, we studied sex chromosome effects on cardiac aging.

Heart weight increased from 3 to 24 months (males + 35%, females + 29%). In males, 75% of this increase had occurred at 12 months; in females, only 30%. Gonadectomy of mice at 12 months blocked cardiac hypertrophy in both sexes during the second year of life. The dosage of the X chromosomes did not influence cardiac growth in young and older mice.

We performed an RNA sequencing study in young and old mice. We identified new highly expressed genes modulated during aging (*Bdh*, *Myot, Cpxm2*, and *Slc38a1*). The myocardial exome in older animals displayed few differences related to the animal's sex or the presence or absence of sex steroids for a year.

We show that the morphological evolution of the heart depends on the biological sex via gonadal sex hormone actions. The myocardial exome of old male and female mice is relatively similar. Our study emphasizes the need to consider sex steroid effects in studying cardiac aging.

## INTRODUCTION

Biological sex differences in cardiac phenotypes are frequently recognized from clinical studies for various cardiovascular diseases. At the same time, many differences in outcomes arise from gender-related factors, just as many arise from factors related to biological sex [1].

A large body of data indicates that biological sex is directly and significantly related to differences in cardiovascular traits. These differences originate from intrinsically distinct phenotypic starting points that precede age-related factors, combined with divergent trajectories that seem mediated by sex-related differences in the response to various risk factors [1].

Heart failure (HF) is defined by the inability of the heart to pump (HF with reduced ejection fraction) or by the failure of the heart to relax, resulting in reduced filling but with preserved ejection fraction (HFpEF). Approximately 50% of HF patients have HFpEF, and more than 60% of them are women, most of them post-menopausal [2]. There is a need for a better understanding of the underlying mechanisms of HFpEF, and several new preclinical models in rodents have been proposed [3–5]. Considering that HFpEF is a condition affecting mainly post-menopausal older women, we must investigate more thoroughly the effects of a loss of gonadal steroids in aging animal models to understand this syndrome and to design improved therapy or interventions [6]. Considering their relatively short lifespan, mice and rats can be practical models to study the combined effects on the heart of aging and the loss of sex steroids.

In response to hemodynamic stress, cardiac hypertrophy develops, with women presenting more frequently with smaller LV diameter and ejection parameter preservation than men [7–9]. Animal studies also show that females maintain better systolic function and have smaller LV dimensions [9, 10]. Studies have shown that the lack of estrogens enhances LV hypertrophy, whereas menopausal hormone therapy and treatment with 17β-estradiol (E2) prevent the development of LV hypertrophy in postmenopausal women and ovariectomized animals, respectively [11–16].

After menopause, women lose protection conferred by estrogens against heart diseases. In aging women, heart disease incidence increases, but sex differences in their manifestations persist [17]. Several explanations have been suggested. Estrogen imprinting may be present years after menopause. Local production of estrogens in peripheral tissues can perpetuate this sexual dimorphism. Morphological and physiological sex differences may be permanently ingrained irrespective of the presence or absence of estrogens. Another possible explanation that has received very little attention so far is the effects of sex chromosomes (SCE). The X chromosome is present in both males and females. Most X genes are expressed equally in XX and XY cells since one copy of X-linked genes is transcriptionally silenced in XX cells. Some X-linked genes can escape this silencing, implying that doubling these genes could cause phenotypical differences. Several of these “escapees” control gene expression, mRNA splicing, mRNA export and cellular signalling [18–21]. These and the other genes they control could be involved in some of the sex differences observed in heart diseases. Powerful sex hormone effects can mask phenotypical differences linked to SCEs. If estrogens were the only factors for sex differences in ischemic heart disease in humans, one could expect a difference only before menopause [22]. Sex differences in ischemic heart disease incidence are present throughout life. The persistence of estrogen effects in women or androgen action in men may cause them. We recently reported that XY and XX sex chromosome complements lead to slight differences in LV remodelling after metabolic-hypertensive stress (Angiotensin II + high-fat diet) in four-core genotype mice [23].

Here, we studied the contribution of aging, sex steroids, and sex chromosomes to cardiac morphology and function in mice of both sexes and proposed a new preclinical model for the study of cardiac health in older women.

Studying menopause in rodents has essentially relied on three models, namely normal aging, ovariectomy and chemically induced ovarian failure using 4-vinyl cyclohexene diepoxide or VCD. The last model is especially useful to study the peri-menopausal period since ovarian failure happens over an extended period, as in women [23]. Recently, Troy and collaborators used this VCD approach to study the effects of menopause in the L-NAME + high-fat diet HFpEF mouse model [3, 5]. They did not observe clear effects of menopause in their animals [24].

In this study, since we focused on the long-term (10 to 12 months) effects of loss of sex steroids on heart morphology, function, and myocardial gene expression in either young or mature female mice, we chose to use ovariectomy. In parallel, we studied the effects of biological sex, sex chromosomes, aging, and the timing of the loss of gonadal sex steroids (at 7 or 52 weeks of age). We show that aging in mice is associated with sex-specific cardiac growth/hypertrophy patterns and that these patterns are influenced more by sex steroids than by sex chromosomes. Using bulk left ventricle RNA sequencing, we also identified several new genes associated with cardiac aging.

## MATERIALS AND METHODS

### Animals

C57BL6/J male and female mice were purchased from Jackson Laboratory (Bar Harbor, ME, USA) or the aging colony of the Quebec Aging Research Network (RQRV) and kept for up to 24 months of age. They were housed on a 12 h light/12 h dark cycle with free access to chow and water.

Four-core genotypes (FCG) (B6.Cg-Tg(Sry)2Ei Srydl1Rlb/ArnoJ) breeders were purchased from Jackson Laboratory. These mice had already been on a C56Bl/6J background for at least eighteen generations. The breeding strategy consisted of mating a C57Bl6/J female with an XY Srydl1Rlb Tg(Sry)2Ei male [25, 26]. The progeny of this cross was either XX normal females (+/+), XY phenotypic females, XX phenotypic males or XY phenotypic fertile males (Sry/Tg(Sry2)). The four possible genotypes in FCG mice were characterized as follows: After gonadectomy, Xist expression by quantitative PCR was determined in the ovaries of females to discriminate between XY and XX animals. The size of testicles was used to identify XX males (small; about half of the standard size) and XY males (normal). The genotype was confirmed in all animals by measuring *Xist* expression in the left ventricle (LV) after euthanasia. Sequences of Xist primers were forward, TAA GGA CTA CTT AAC GGG CT and reverse, TAC TCA GAC ATT CCC TGG CA.

The protocol was approved by the Université Laval’s animal protection committee and followed the recommendations of the Canadian Council on Laboratory Animal Care (#2019-075 and #2020-603). This study was conducted according to ARRIVE guidelines.

### Experimental design

**Figure 1:**
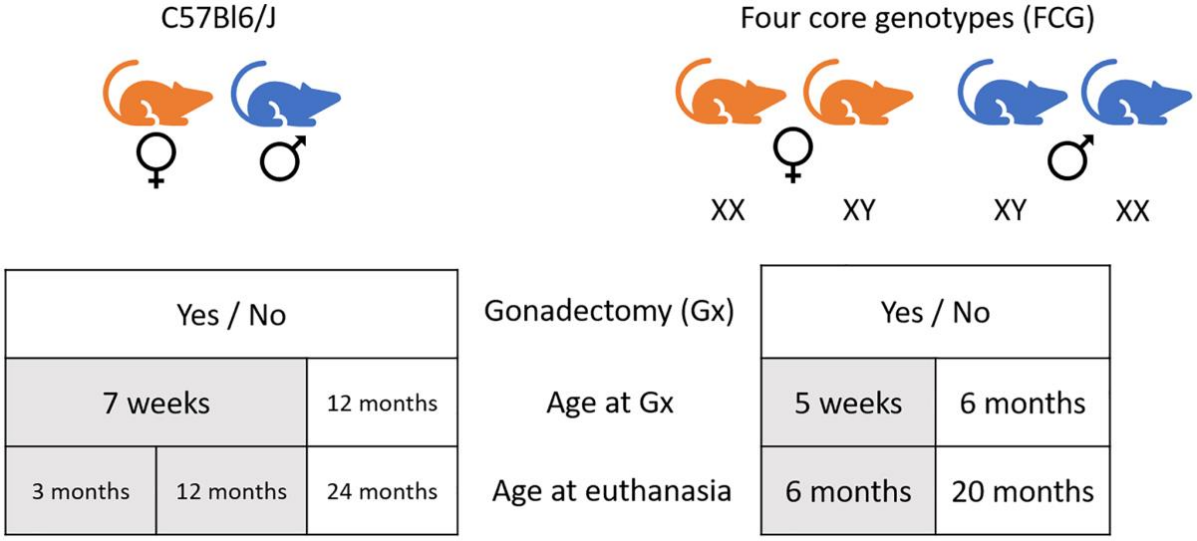
Schematic representation of the experimental design of the study.

The experimental design is schematized in Figure 1.

Three-month-old mice: Sixteen 7-week-old mice (8 males and eight females) were used to form the young control groups. These mice were euthanized at the age of three months.

Twelve-month-old C57Bl6/J mice: Thirty 7-week-old mice (14 males and 16 females) were randomly distributed between 4 groups. Half of the animals were gonadectomized (Gx) at seven weeks [27].

Twenty-four-month-old C57Bl6/J mice: Forty-one 12-month-old mice (24 males and 17 females) were randomly distributed between 4 groups: males and females, Gx at 12 months or not.

Twenty-nine FCG 5-week-old mice were Gx and distributed between the four possible genotypes (Males: XY and XX, females: XX and XY). All mice had an echocardiography exam at two months and the day before euthanasia at six months of age.

Forty-six FCG mice were Gx at six months and distributed between the four genotypes. All mice had an echocardiography exam at 6, 12 and 20 months the day before euthanasia.

Animals were monitored daily by experienced technicians for health and changes in behaviour during the protocol. Mice display signs associated with poor prognosis of quality of life or specific signs of severe suffering or distress. Among those signs are significant loss or gain of weight, palpable tumours and grooming habits. All older mice over 12 months of age had available in their cage a running saucer (Innowheel™, Innovive, Billerica, MA, USA) as an environmental enrichment to avoid unwanted behaviours such as barbering.

Euthanasia was performed by total exsanguination of isoflurane-anesthetized animals, followed by cervical dislocation. Hearts were quickly removed, rinsed in phosphate-buffered saline, and weighed. The left atrium was then dissected and weighed. Lungs were also weighed.

### Echocardiography

Unless specified above, all mice have an echocardiography (Echo) exam performed the day before euthanasia. The same investigator, blinded for mouse identification, acquired Echo images on a Vevo 3100 imaging system (VisualSonics, FujiFilm, Toronto, Canada). Transthoracic echocardiography was performed using a 40 MHz image transducer (MX550S) as previously described [27, 28]. Briefly, animals were anesthetized using isoflurane. The concentration of isoflurane was maintained around 2.5–3.5%. Images were taken from the LV parasternal long axis (PSLAX; B-mode and M-mode) view. PSLAX views (at least three consecutive heartbeats) were used to delineate the LV trace end-diastole and end-systole to obtain the averaged measurements of heart rate (HR), end-diastolic volume (EDV), end-systolic volume (ESV), stroke volume (SV), ejection fraction (EF), and cardiac output (CO) with the VevoLab software (VisualSonics). The wall thickness (septal, IVSW; posterior, PW) and internal end-diastolic and end-systolic diameters (EDD and ESD) of the LV were measured in at least three consecutive cardiac cycles in PSLAX M-mode that were averaged. Fractional shortening (FS), relative wall thickness (RWT) and corrected LV mass were calculated using the Vevo Lab software. Images were also taken in pulse wave Doppler and tissue Doppler modes to measure blood velocity through the mitral valve and velocity of the mitral annulus, respectively. E wave, A wave and E/A ratio were estimated using the VevoLab software.

### Histological analysis

Tissue preparation. OCT-embedded frozen cardiac tissue sections (long axis) were cut 10 μm thick and fixed on a microscope slide. LV sections were fixed using Bouin’s solution overnight, and the nuclei were stained using Wiegert’s hematoxylin for 10 minutes. Fibrosis was then stained using Picrosirius Red dye for 1 hour. Images of each section of the LV (interventricular septal wall (IVS), apex, posterior or free wall, and LVPW) were acquired using a wide-field microscope (Zeiss). Each of these sections was then analyzed using GIMP (GNU Image manipulation program; https://www.gimp.org/). Both microscopy and image analyses were performed by investigators who were blinded for mouse identification.

LV fibrosis assessment. Three sections of the LV (septum, apex, and free wall) were independently analyzed, and the mean percentage of interstitial fibrosis of the three sections was determined. Briefly, LV fibrosis of each section was measured by calculating the ratio of stained (red) pixels to the total number of pixels representing cardiomyocytes and fibrosis. The number of pixels corresponding to white sections (holes in the tissue) was measured and deducted from the total number of pixels of the image to get the total number of pixels representing the myocardium. Measures were acquired using the “by colour select” tool in the software.

Cardiomyocyte cross-sectional area (CSA). When acquiring the images, an LV image on which cardiomyocytes were easily visible and in the “en face” position was explicitly taken for cardiomyocyte CSA measurements. At least fifteen cardiomyocytes were measured for the three sections described above. The mean was calculated for each sample. Cardiomyocyte CSA was measured in pixels using the “free select” tool in the software. The pixel area was then converted to μm^2^ by multiplying the number of pixels for one cardiomyocyte by the size in μm^2^ of a single pixel. The microscope software automatically calculates a single pixel’s width and length in μm.

### RNA preparation for mRNA sequencing

Total RNA was extracted from cardiac tissue samples using TRI Reagent (Sigma, Mississauga, Ontario, Canada). RNA from LV was removed for 3 to 4-month-old males and females, 12-month-old males and females, and all 24-month-old experimental groups. Samples were then treated with DNase I (RapidOut DNA removal kit, Thermo Fisher Scientific, Waltham, MA, USA), and RNA integrity was analyzed with the Agilent 2100 Bioanalyzer and RNA 6000 Nano kit.

### Bulk mRNA-sequencing

LV samples, three to four pools of total RNA from two mice for each experimental group, were used to create libraries using Stranded mRNA Prep and Ligation kit (Illumina, San Diego, CA, USA) following the manufacturer’s recommendations. Libraries were analyzed using the Agilent 2100 Bioanalyzer and DNA 1000 kit. All samples were then quantified using the KAPA Library Quantification Kit (Kapa Biosystems). The average size of libraries was 335 base pairs. Indexed libraries were pooled and sequenced on the Illumina NextSeq 2000 (paired-end 150 bp) using sequencing-by-synthesis chemistry v4 according to the manufacturer’s protocols. We averaged 8.0 × 10^7^ (3.7 to 15.3 × 10^7^) paired-end reads, with an average of >95% of the reads achieving a quality score equal to or greater than Q30. Illumina BaseSpace RNA-Seq Alignment (STAR) RNA-Seq Differential Expression (DESeq2) software for sequence alignments and identifying differentially expressed genes (DEG) using the Mus musculus UCSC 10 mm reference genome. The DEGs were determined by adjusting the *p*-value for multiple tests using Benjamini–Hochberg correction with false discovery rate (FDR) ≤0.05 and Log2 fold change (Log2FC), |Log2 FC| ≥1.0. Gene set enrichment analysis was performed using the Panther GO Enrichment Analysis (https://geneontology.org/) [29–31]. A differentially expressed genes list (FDR <0.05 and |Log2FC| ≥1.0) was used to perform this analysis. Significant GO terms were determined using Fischer’s Exact test using Benjamini–Hochberg correction with FDR.

### Statistical analysis

All data are expressed as mean ± standard error of the mean (SEM). Intergroup comparisons were conducted using the Student’s *T*-test using GraphPad Prism 10.0 (GraphPad Software Inc., La Jolla, CA, USA). Comparisons of more than two groups were analyzed using one-way or two-way ANOVA and Holm-Sidak post-test. *P* < 0.05 was considered statistically significant.

## RESULTS

### The heart gradually gains mass during life in female mice

**Figure 2:**
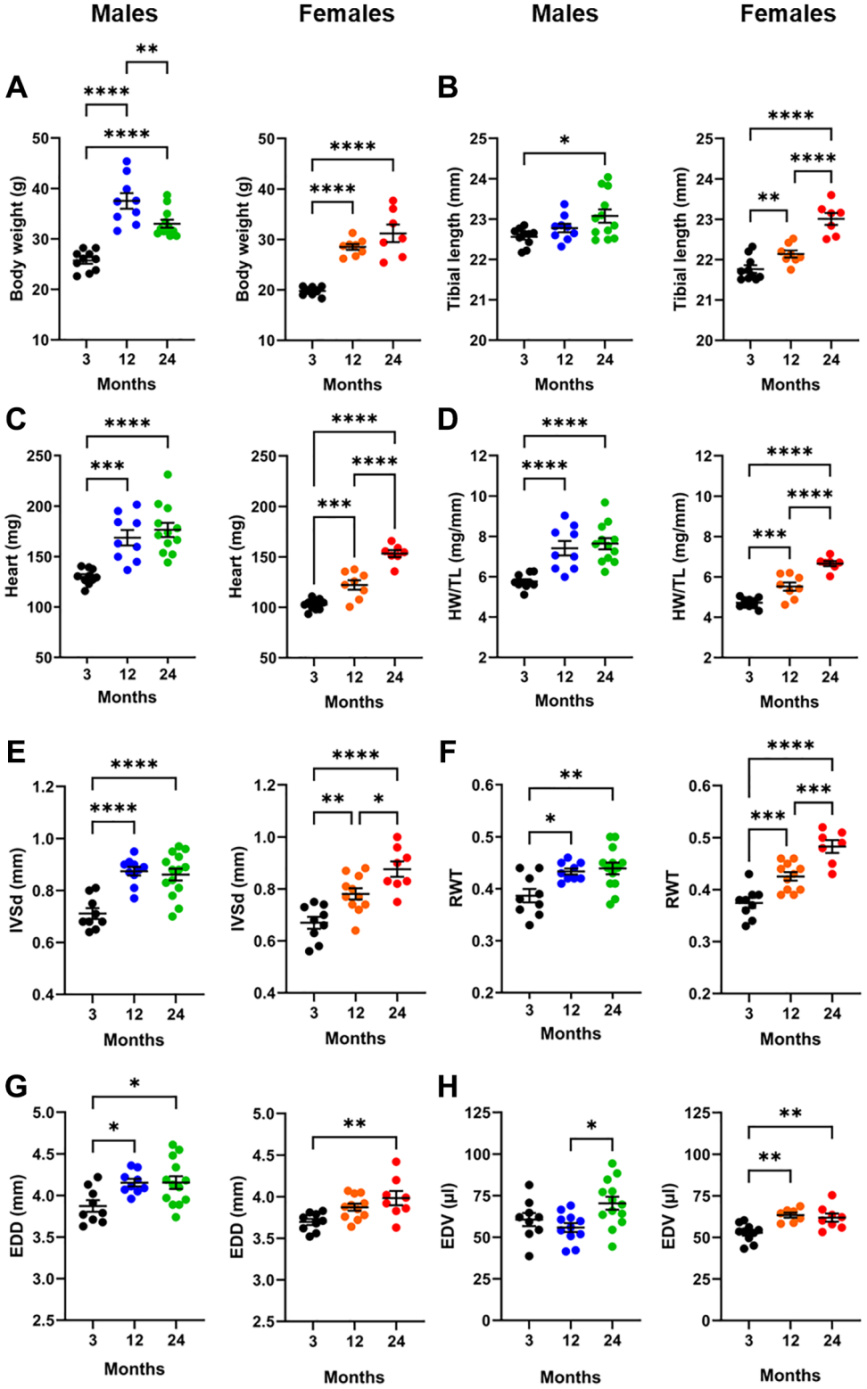
**Effects of aging on the heart. For each panel, graphs of males (blue and green) and females (orange and red) are represented side by side.** (**A**) Body weight, (**B**) Tibial length, (**C**) Heart weight, (**D**) indexed heart weight for tibial length (HW/TL), (**E**) Diastolic thickness of the interventricular septum (IVSd) by echo, (**F**) End-diastolic LV diameter (EDD), (**G**) Left ventricular relative wall thickness (RWT) and (**H**) End-diastolic LV volume (EDV). Data are represented as mean +SEM. One-way ANOVA followed by Holm-Sidak post-test. ^*^p < 0.05, ^**^p < 0.01, ^***^p < 0.001 and ^****^p < 0.0001 between indicated groups.

Groups of male and female C57Bl6/J mice were sacrificed at the age of 3 (young), 12 (adult) and 24 months (old). As illustrated in Figure 2A, young male mice gained weight during the first ten months of follow-up (+46%) and then lost 12% of their body weight during the second year. In females, the 44% increase in body weight during the first year of life was followed by relatively stable values. We used tibial length as a surrogate of body growth. For both sexes, tibial growth was present up to 24 months. The overall growth was less in males (2.3%) than in females (5.7%) (Figure 2B). Body weight and tibial length were similar between male and female mice at 24 months. Heart weight (HW) in males increased by 30% (22% for indexed HW (HW/TL)) until 12 months, then remained stable after that. In females, HW gained 19% (17% for indexed HW) at 12 months and an additional 17% (21% for iHW) in the second year (Figure 2C, 2D).

### Aging is associated with left ventricle concentric remodelling

An echo exam was performed the day before the euthanasia at 3, 12 and 24 months. Detailed echo data are described in Tables 1 and 2. In males, as illustrated in Figure 2E, end-diastolic interventricular septum (IVSd) thickness was markedly increased in adults compared to younger animals (+20%) and remained stable until 24 months. In females, the IVSd thickness increase was gradual up to 24 months (+17% at 12 months and +12% at 24 months). End diastolic LV diameter (EDD) was larger in males at 12 months (+7%) and remained unchanged in older animals. EDD had increased by 8% in females in two-year-old mice compared to young females (Figure 2F). In males, relative wall thickness (RWT), an index of LV remodelling, increased until 12 months and then remained unchanged. In females, the RWT increase was gradual over two years. Interestingly, RWT at 24 months was significantly higher in females than males (*p* = 0.024), suggesting a more concentric LV remodelling in females (Figure 2G).

**Table 1:** Echocardiography (Echo) data from young and aging male mice.

**Parameter**	**M3 *N* = 10-12**	**M12 *N* = 12**	**M12 Gx *N* = 12**	**M24 *N* = 13-14**	**M24 Gx *N* = 10**
***M-Mode***					
EDD, mm	3,9 ± 0,05	4,2 ± 0,08^**^	3,8 ± 0,04^§§§^	4,2 ± 0,11^**^	4,2 ± 0,06
ESD, mm	2,7 ± 0,08	2,8 ± 0,10	2,6 ± 0,07	2,9 ± 0,09	2,8 ± 0,06
PW, mm	0,79 ± 0,014	0,95 ± 0,024^****^	0,80 ± 0,009^§§§§^	0,95 ± 0,025^****^	0,91 ± 0,020
IVS, mm	0,72 ± 0,020	0,88 ± 0,016^****^	0,75 ± 0,014^§§§§^	0,86 ± 0,022^****^	0,86 ± 0,018
RWT	0,39 ± 0,010	0,43 ± 0,012^*^	0,40 ± 0,007^§^	0,43 ± 0,013^*^	0,43 ± 0,007
LV mass, mg	92 ± 1.8	120 ± 5.3^**^	90 ± 1.4^§§§^	127 ± 3.9^***^	100 ± 1.6^§§^
***Simpson’s***					
SV, mm	30,3 ± 0,97	41,3 ± 1,35^***^	27,0 ± 1,61^§§§§^	39,3 ± 2,20^***^	36,8 ± 1,87
EF, %	56,8 ± 2,54	64,6 ± 1,61^*^	57,5 ± 1,70^§§^	54,5 ± 1,65	56,9 ± 1,60
HR, bpm	445 ± 15,3	490 ± 13,4	449 ± 9,5^§^	473 ± 12,1	503 ± 5,1
CO, ml/min	13,4 ± 0,48	20,3 ± 0,85^****^	12,1 ± 0,65^§§§§^	18,6 ± 1,15^***^	18,5 ± 1,05
EDV, μl	54,3 ± 2,32	64,5 ± 3,28	47,2 ± 2,69^§§§^	72,8 ± 4,30^**^	64,7 ± 2,89
ESV, μl	24,0 ± 2,43	23,2 ± 2,30	20,2 ± 1,63	33,5 ± 2,67^*^	28,0 ± 1,64
***Doppler***					
E, mm/s	629 ± 14,7	664 ± 15,2	637 ± 14,6	662 ± 30,3	646 ± 20,7
A, mm/s	398 ± 11,6	434 ± 16,1	405 ± 13,9	426 ± 21,3	441 ± 15,9
E/A	1,59 ± 0,051	1,54 ± 0,034	1,58 ± 0,025	1,56 ± 0,041	1,48 ± 0,054
E’, mm/s	−25,3 ± 0,98	−28,6 ± 1,25	−27,0 ± 1,08	−29,9 ± 1,82	−30,2 ± 1,15
A’, mm/s	−16,2 ± 0,80	−17,7 ± 0,52	−17,6 ± 0,71	−19,2 ± 1,42	−19,5 ± 0,58
E/E’	−24,9 ± 1,08	−23,6 ± 0,90	−24,0 ± 1,09	−22,1 ± 0,66	−21,5 ± 0,60
E’/A’	1,58 ± 0,068	1,61 ± 0,047	1,54 ± 0,055	1,60 ± 0,067	1,56 ± 0,050

Left ventricle parameters were measured in male (M) mice at three time points, namely in young (3 months; 3), adult (12 months; 12), orchiectomized (Gx) or not, and old (24 months; 24) Gx or not animals. Echo exams were performed as described in the Methods section. Abbreviations: EDD: end-diastolic LV diameter; ESD: end-systolic LV diameter; PW: diastolic posterior wall thickness; IVS: diastolic inter-ventricular septum thickness; RWT: relative wall thickness; and LV: left ventricle; Using Simpson’s method; SV: Stroke volume; EF: Ejection fraction; HR: Heart rate; CO: Cardiac output; EDV: end-diastolic volume and ESV: end-systolic volume. Using pulsed-wave and tissue Doppler. E: E wave; A: A wave; E’: E’ wave and A’: A’ wave. Results are expressed as the mean ± standard error of the mean (SEM) from the indicated number of animals. One-way ANOVA analysis and Holm-Sidak post-test using data in 3-month-old males as controls were used to analyze statistical differences between 12-month-old and 24-month-old mice. ^*^p < 0.05, ^**^p < 0.01 and ^****^p < 0.0001. Student’s *T*-test was used to analyze statistical differences between Gx groups and their age-corresponding non-Gx group: ^§^*p* < 0.05, ^§§^*p* < 0.01, ^§§§^*p* < 0.001 and ^§§§§^h < 0.0001.

**Table 2:** Echocardiography (Echo) data from young and aging female (F) mice.

**Parameter**	**F3 *N* = 11–12**	**F12 *N* = 12**	**F12 Gx *N* = 11–12**	**F24 *N* = 7–8**	**F24 Gx *N* = 8**
***M-Mode***					
EDD, mm	3,7 ± 0,03	3,8 ± 0,07	3,9 ± 0,08	4,0 ± 0,09	3,8 ± 0,04
ESD, mm	2,6 ± 0,06	2,6 ± 0,08	2,7 ± 0,10	2,6 ± 0,08	2,6 ± 0,08
PW, mm	0,74 ± 0,022	0,84 ± 0,018^****^	0,92 ± 0,022^§^	0,97 ± 0,045^****^	0,86 ± 0,022^§^
IVS, mm	0,67 ± 0,026	0,76 ± 0,022^***^	0,83 ± 0,023	0,88 ± 0,030^****^	0,77 ± 0,016^§^
RWT	0,38 ± 0,013	0,42 ± 0,011^*^	0,45 ± 0,016	0,47 ± 0,021^***^	0,42 ± 0,012^§^
LV mass, mg	85 ± 1.4	106 ± 2.4^*^	107 ± 5.0	117 ± 3.8^***^	102± 4.6^§^
***Simpson’s***					
SV, mm	26,1 ± 1,25	35,8 ± 1,93^***^	33,4 ± 1,78	36,8 ± 2,31^**^	31,8 ± 1,09
EF, %	57,6 ± 1,91	65,8 ± 1,64^*^	63,2 ± 1,55	59,4 ± 2,37	56,1 ± 1,78
HR, bpm	448 ± 21,5	482 ± 6,1	477 ± 7,0	480 ± 30,0	489 ± 6,2
CO, ml/min	11,8 ± 0,89	17,2 ± 0,85^***^	15,9 ± 0,77	16,3 ± 2,15^**^	15,5 ± 0,46
EDV, μl	45,3 ± 1,34	54,8 ± 3,22	52,9 ± 2,45	61,9 ± 2,46^***^	56,9 ± 1,75
ESV, μl	19,2 ± 1,00	19,0 ± 1,73	19,5 ± 1,25	25,1 ± 1,56	25,1 ± 1,55
***Doppler***					
E, mm/s	583 ± 20,8	640 ± 10,0	662 ± 22,1	614 ± 41,9	626 ± 18,2
A, mm/s	385 ± 14,8	414 ± 8,0	433 ± 18,6	396 ± 26,1	418 ± 10,0
E/A	1,53 ± 0,054	1,55 ± 0,026	1,54 ± 0,054	1,55 ± 0,046	1,51 ± 0,059
E’, mm/s	−26,4 ± 1,29	−28,2 ± 0,73	−26,9 ± 0,67	−29,6 ± 1,99	−28,2 ± 0,96
A’, mm/s	−18,1 ± 0,78	−16,1 ± 0,59	−17,9 ± 1,00	−19,5 ± 2,02	−18,1 ± 0,56
E/E’	−22,4 ± 0,72	−22,8 ± 0,56	−24,4 ± 1,04	−20,9 ± 1,40	−22,3 ± 0,50
E’/A’	1,46 ± 0,046	1,77 ± 0,074	1,54 ± 0,067^§^	1,56 ± 0,096	1,56 ± 0,051

Left ventricle parameters were measured in male (M) mice at three time points, namely in young (3 months; 3), adult (12 months; 12), ovariectomized (gx) or not, and old (24 months; 24) Gx or not animals. Abbreviations: EDD: end-diastolic LV diameter; ESD: end-systolic LV diameter; PW: diastolic posterior wall thickness; IVS: diastolic inter-ventricular septum thickness; RWT: relative wall thickness; and LV: left ventricle; SV: Stroke volume; EF: Ejection fraction; HR: Heart rate; CO: Cardiac output; EDV: end-diastolic volume; ESV: end-systolic volume. E: E wave; A: A wave; E’: E’ wave; A’: A’ wave. Results are expressed as the mean ± standard error of the mean (SEM) from the indicated number of animals. One-way ANOVA analysis and Holm-Sidak post-test using data in 3-month-old females as controls were used to analyze statistical differences between 12-month-old and 24-month-old mice ^*^p < 0.05, ^**^p < 0.01 and ^****^p < 0.0001. Student’s *T*-test was used to analyze statistical differences between Gx groups and their age-corresponding non-Gx group: ^§^*p* < 0.05, ^§§^*p* < 0.01, ^§§§^*p* < 0.001 and ^§§§§^h < 0.0001.

End diastolic LV volumes (EDV) followed a different trend. In males, EDV was stable during the first year and then increased in 24-month-old mice (+26%). In females, a 20% increase in EDV was observed up to 12 months, remaining unchanged (Figure 2H). Detailed echocardiography data are listed in Tables 1 and 2.

### Sex and age differences in the heart response to losing gonadal steroids

We then investigated how gonadectomy (Gx), either in young (7-week-old) or adult mice (12 months), would influence heart growth and LV morphology. We compared 12- and 24-month-old male and female mice after losing gonadal sex steroids for 10 and 12 months, respectively, to same-age control animals.

**Figure 3:**
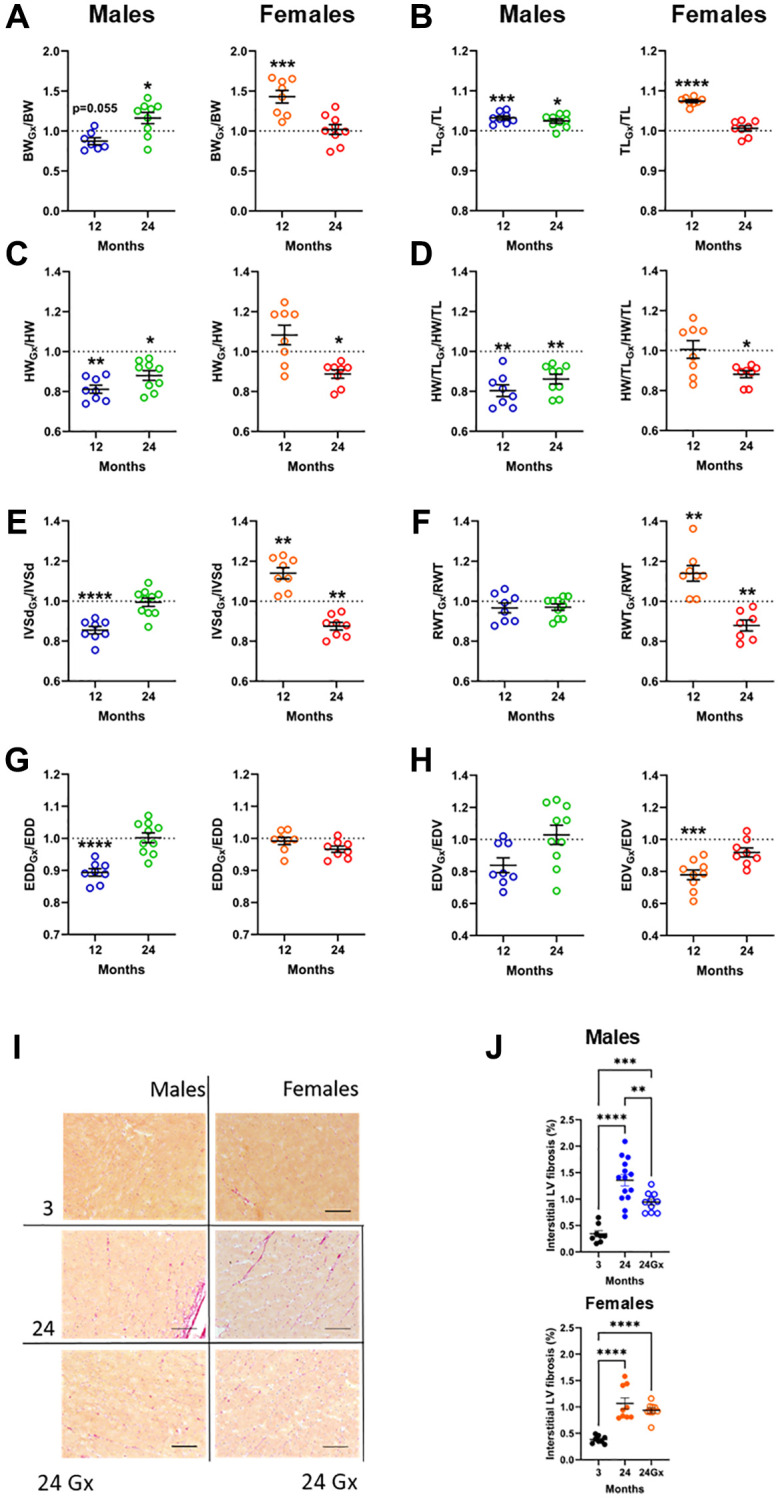
**Loss of gonadal steroid hormones in young and adult mice and its effects on the heart later in life.** (**A**–**H**) Results are illustrated as a ratio of individual values of indicated parameters in gonadectomized (Gx) animals on the mean value for this parameter from a group of age-matched controls. The dotted line indicates a ratio of 1 or the absence of difference between Gx and non-Gx animals. Blue and green (males) and orange and red (females) indicate the ratio in adult (12 months) and old mice (24 months). (**A**) Body weight, (**B**) Tibial length, (**C**) Heart weight, (**D**) indexed heart weight for tibial length (HW/TL), (**E**) Diastolic thickness of the interventricular septum (IVSd) by echo, (**F**) End-diastolic LV diameter (EDD), (**G**) Left ventricular relative wall thickness (RWT) and (**H**) End-diastolic LV volume (EDV). (**I**) Representative pictures of picrosirius-stained LV sections for the indicated groups. (**J**) Interstitial myocardial fibrosis in aging male and female mice, intact or Gx. Data are represented as mean +SEM. (**A**–**H**) Student *T*-test comparison with age-matched controls. (**J**) One-way ANOVA followed by Holm-Sidak post-test. ^*^p < 0.05, ^**^p < 0.01, ^***^p < 0.001 and ^****^p < 0.0001.

As illustrated in Figure 3A, 3I, the body weight (BW) of 12-month-old ovariectomized (Gx) males tended to be lower than for control adult males, whereas, in old males, BW was higher in Gx animals at 24 months. In females, Ovariectomy (Gx) of young female mice resulted in a higher BW 10 months later, whereas late Gx did not affect this parameter. Gx increased tibial length for both males and females at 12 months. Interestingly, this effect was also present in old Gx males (Figure 3B, 3I). The evolution of body weight in older mice after Gx is illustrated in Supplementary Figure 1A. It shows apparent sex differences. Cardiac growth was reduced in younger Gx males by almost 20%, whereas Gx had no effects on HW or indexed HW (iHW). In 2-year-old males or females, loss of sex hormones at 12 months resulted in lower HW compared to age-matched controls (Figure 3C, 3D and Supplementary Table 1).

Using cardiac ultrasound, we studied LV morphology changes caused by Gx. In younger males, LV septal wall thickness and diastolic diameter were reduced by Gx, resulting in a relatively stable RWT and reduced EDV. In older males, Gx had no effect during the second year of life (Figure 3E–3H). Gx increased LV septal wall thickness in younger females without affecting LV diameter. This resulted in increased RWT and a smaller LV chamber, as indicated by the reduced EDV. In older females, Gx slowed the continuing LV concentric remodelling during the second year of life, as evidenced by the decreased IVSd, RWT and EDV (*p* = 0.11) of about 10%. Supplementary Figure 1B illustrates the evolution of LV wall thickness in older male and female mice after Gx. Detailed echocardiography data for Gx animals are shown in Tables 1 and 2.

As illustrated in Figure 3I, 3J, aging was associated with increased interstitial LV fibrosis at 24 months. In males, Gx at 12 months reduced the development of interstitial myocardial fibrosis as observed a year later at 25 months, whereas in females, loss of sex steroids had no effect. At 12 months, interstitial fibrosis was not significantly higher than in young mice (not shown).

### Sex chromosomes have limited effects on cardiac morphological changes associated with aging

To study the effects of sex chromosomes, we used the transgenic four-core genotypes (FCG) mouse line where the sex chromosome complement is dissociated from the sex phenotype. In these mice, the *Sry* gene, which is involved in developing the male phenotype, is no longer located on the Y chromosome but on an autosome (24). This allows male and female XX or XY mice to be obtained.

We studied these mice by echocardiography from 2 months up to 20 months. Mice that were euthanized at six months were gonadectomized (Gx) at the age of 5 weeks to remove the contribution of sex steroids during most of their life. Non-Gx FCG mice of the same age were euthanized as controls. In mice euthanized at 20 months, gonadectomy was performed at the age of 6 months to remove the effects of sex steroids later in life and to simulate, at least for females, menopause. Non-Gx mice were also studied for up to 20 months.

**Figure 4:**
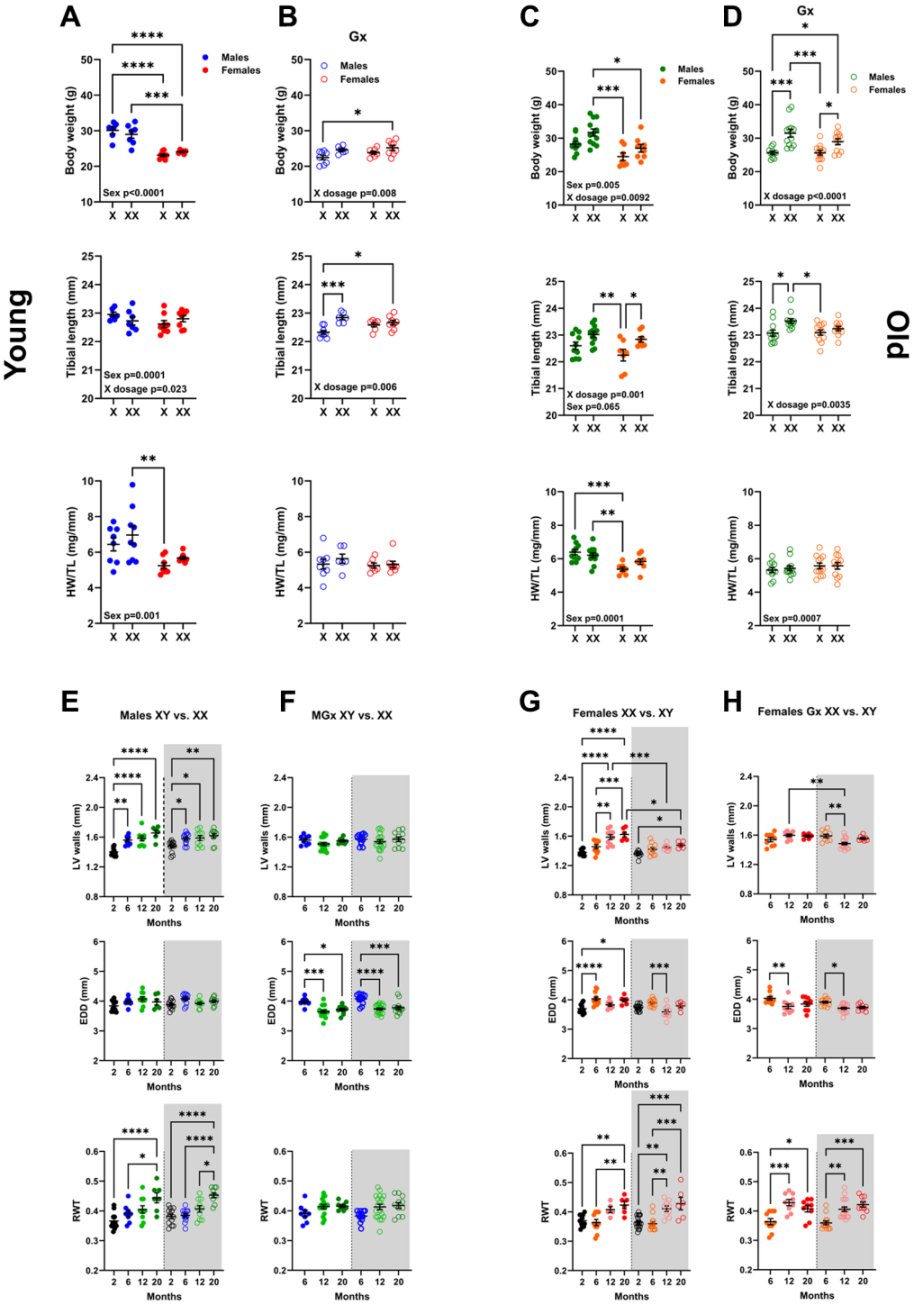
**Sex chromosomes significantly affect body growth but little on cardiac growth and LV remodelling during aging.** Body weight, Tibial length, and indexed Heart weight for tibial length (HW/TL). A and B: Young mice (6 months). (**A**) non-Gx 6-month-old mice with one (X) or two X chromosomes (XX). Phenotypic males (solid blue dots) and females (solid red dots). (**B**) Gx 6-month-old mice (Gx at five weeks). Phenotypic males (blue circles) and females (red circles). (**C**, **D**) Old mice (20 months). (**C**) non-Gx 20-month-old mice. Phenotypic males (solid green dots) and females (solid orange dots). (**D**) Gx 20-month-old mice (Gx at six months). Phenotypic males (green circles) and females (orange circles). Data are represented as mean +SEM. Two-way ANOVA followed by Holm-Sidak post-test. ^*^p < 0.05, ^**^p < 0.01, ^***^p < 0.001 and ^****^p < 0.0001 between indicated groups. Variables are phenotypical sex (Sex) and one or two X chromosomes (X dosage). *P*-values below 0.05 are indicated on the graphs for the two variables. (**E**–**H**) LV wall thickness, End-diastolic LV diameter (EDD) and LV relative wall thickness (RWT) during aging by echocardiography. (**E**) Non-Gx males with one X chromosome (left; solid dots) or two X chromosomes (right; empty dots and gray background). (**F**) Gx males (MGx) at the age of 6 months. (**G**) Non-Gx females with two X chromosomes (left; solid dots) or one X chromosome (right; empty dots and gray background). (**H**) Gx females (FGx) at the age of 6 months. Data are represented as mean +SEM. One-way ANOVA followed by Holm-Sidak post-test. ^*^p < 0.05, ^**^p < 0.01, ^***^p < 0.001 and ^****^p < 0.0001 between indicated groups.

As illustrated in Figure 4A, in 6-month-old non-Gx mice, female animals had smaller bodies and indexed heart weights (HW/TL) than males, as expected. The number of X chromosomes did not affect these parameters. A small contribution of X dosage was observed for body weight and tibial length. Mice with an abnormal sex chromosome complement (XX for males and XY for females) tend to have lower body weight and shorter tibial weight.

In Gx mice, having two X chromosomes resulted in higher body weight and longer tibial length. However, this effect did not translate to indexed heart weight, which remained identical for all four genotypes (Figure 4B).

In older mice at 20 months, X dosage effects were present in both Gx and non-Gx mice. In Figure 4C, non-Gx male mice had higher body weights, but those with two X chromosomes were heavier than their normal XY counterparts. The tibial length was also longer in XX mice. Indexed heart weights remained unchanged in male or female mice, notwithstanding their chromosome complement. In older Gx mice, having two X chromosomes resulted in heavier body weight and longer tibial length, but again, no differences were observed for heart weight (Figure 4D).

We then investigated by echocardiography if sex chromosomes affected LV morphology or if they could influence its remodelling during FCG mice aging. The animals had an echocardiography exam at 2, 6, 12, and 20 months. As illustrated in Figure 4E, in non-Gx males, X dosage did not affect LV wall thickening during aging and the associated concentric LV remodelling as indicated by LV relative wall thickness (RWT). This was also true in Gx males (Figure 4F). In FCG females, LV wall thickening during aging was slowed in XY mice, and this effect of X dosage was surprisingly more evident in non-Gx than in Gx females (Figure 4G, 4H). More complete echo data (at 2 and 20 months) are described in Supplementary Tables 2–5.

### A transcriptomic study of left ventricular genes modulated during aging

**Figure 5:**
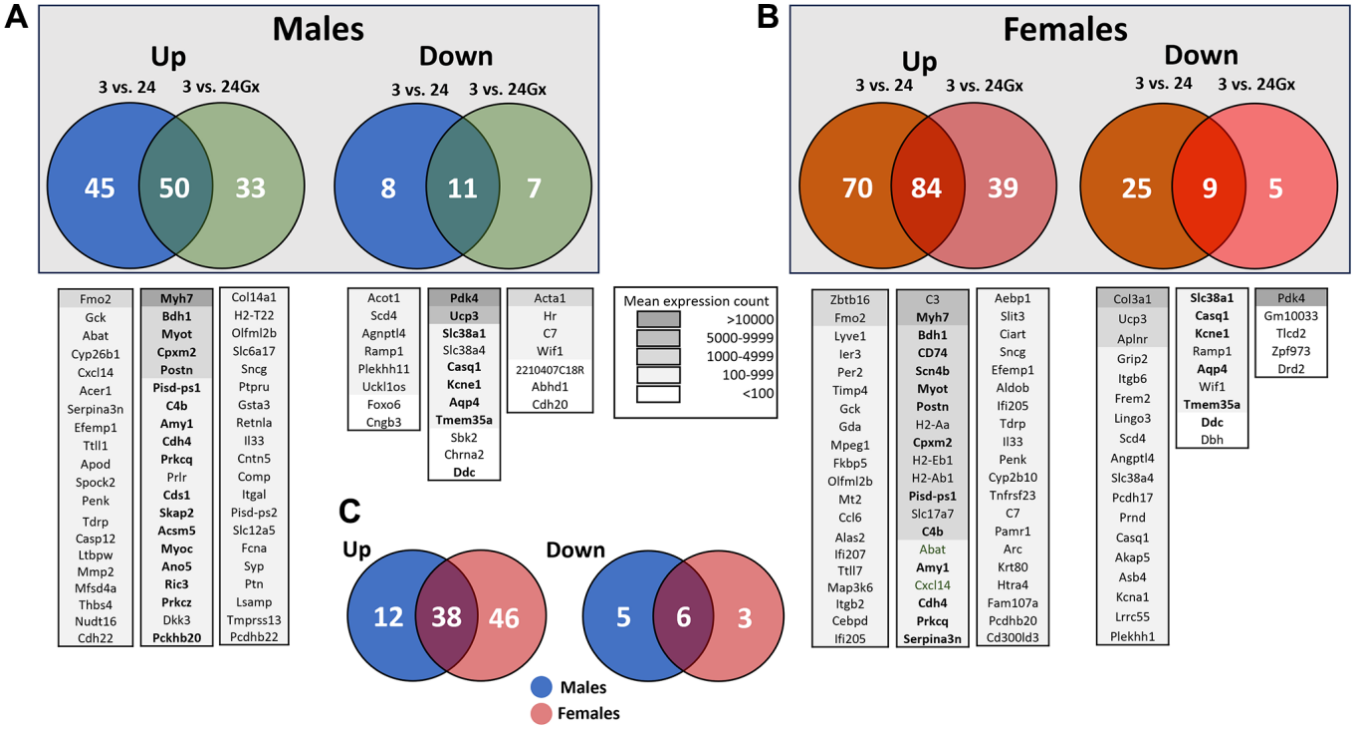
**Transcriptomic changes in the left ventricle induced by aging and gonadectomy.** Differentially expressed genes (DEGs, |Log2 fold change| ≥1) between 3-month-old (3 m) and 24-month-old males (24 m) (**A**) or between 3-month-old and 24-month-old female (**B**) mice, intact or Gx. (**A**) Venn diagrams showing DEGs specific to aging intact or Gx males as well as shared genes either up (left) or down-regulated (right). Venn diagrams showing DEGs specific to aging intact or Gx females and common genes either up (left) or down-regulated (right). Below the Venn diagrams are lists of up to twenty genes ranked by their mean expression levels for each comparison from highest to least expressed. Legend for the background colouring of the gene list based on mean expression count is illustrated. In boldface are listed genes that are common between males and females. (**C**) Venn diagrams for the common upregulated genes (left) and downregulated genes (right) by aging between males and females. Only genes having a mean normalized count over 50 from RNA-Seq data were considered.

We performed a bulk RNA sequencing (RNA-Seq) analysis from a piece of the LV mid-posterior wall from mice of both sexes at 3 (young) and 24 months (old; Gx or not). As mentioned above, we defined DEGs as genes having a |Log2 FC| ≥1.0. As illustrated in Figure 5A, 5B, 154 LV genes in males (Non-Gx and Gx) and 232 genes (Non-Gx and Gx) in females met this criterion when comparing 3-month-old mice to 24-month-old ones. Over 80% of these modulated genes were upregulated in older animals. We then identified the shared genes upregulated during aging in either male or female mice at 24 months of age (non-Gx vs. Gx) compared to young animals. Venn diagrams in Figure 5A, 5B list the 20 most-expressed genes for each comparison. Male and female mice shared 38 up-regulated genes by aging and six down-regulated (Figure 5C).

**Figure 6:**
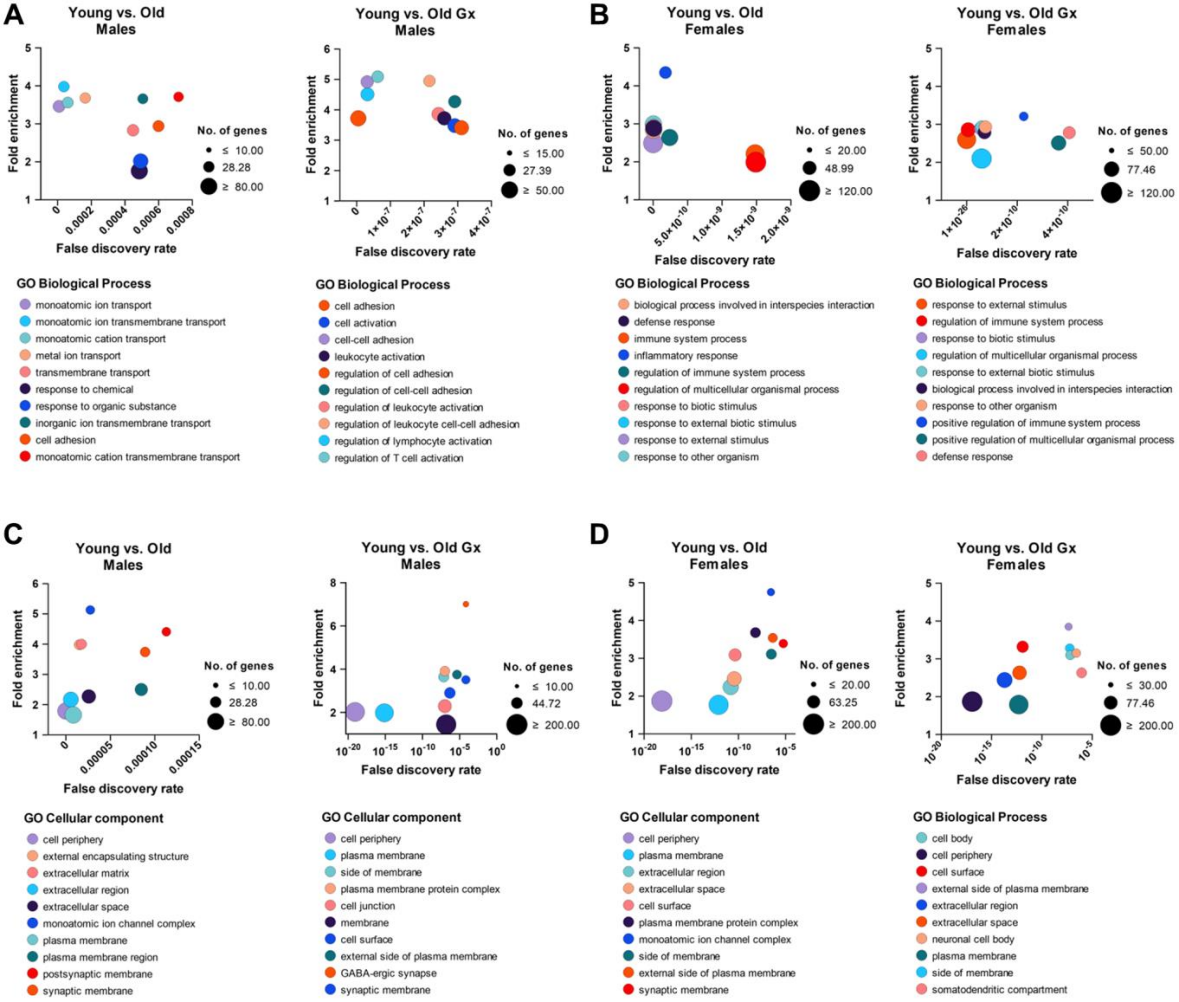
Gene ontology (GO) lists biological processes (**A**, **B**) and cellular components (**C**, **D**) from differentially expressed left ventricle genes between young and older mice represented as bubble plots. All genes significantly modulated (|Log2 fold change| ≥1) were included. The list is limited to the top 10 processes (listed below the graph) having the lowest false discovery rate. Number (Nb) of genes included for each category (bubble size, legend on the right of each graph), fold enrichment over expected representation (Y-axis) and false discovery rate (X-axis).

LV aging highlighted the enrichment of differentiated expressed genes (DEG) for several biological processes and cellular components. Figure 6A, 6B describes a list of biological processes where enrichment of DEG is observed for comparison between young and old mice (Gx or not). Interestingly, in males, only one biological process is shared between intact and Gx old males compared to their young counterparts. Ion transport processes are more present in non-Gx males, whereas, in Gx males, it was linked to inflammation. In females, biological processes were similar between Gx and non-Gx old female mice. The cellular components described in Figure 6C, 6D for male and female old mice were associated with the cell membrane and the extracellular region. Only a few genes were differentially expressed when comparing old mice groups (Gx vs. non-Gx), as listed in Supplementary Tables 6–8.

**Figure 7:**
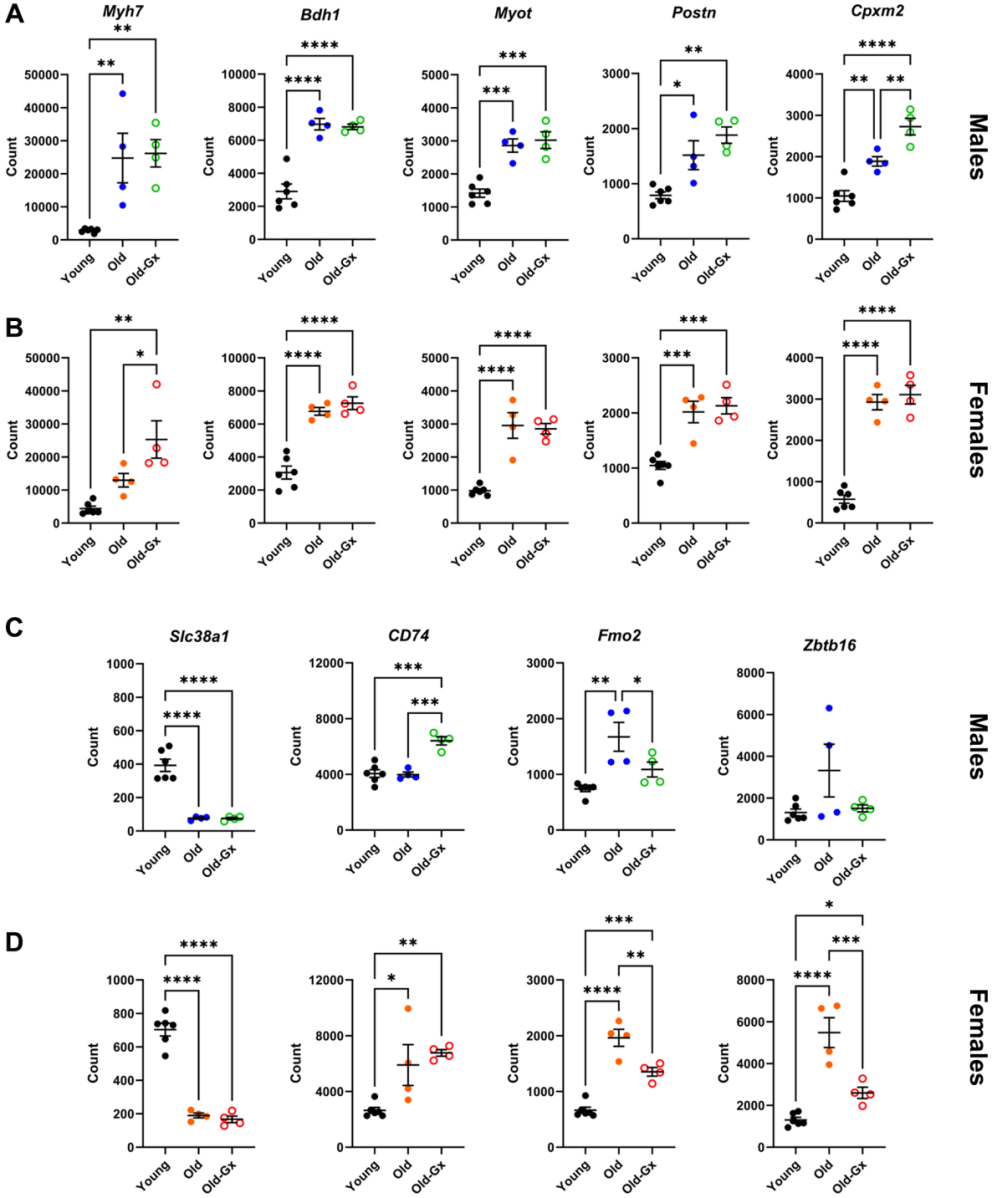
Gene expression of common LV genes up-regulated during cardiac aging in male (**A**) and female (**B**) mice. *Myh7*, Myosin heavy chain beta, *Bdh1*, 3-Hydroxybutyrate Dehydrogenase 1, *Myot*, Myotilin, *Postn*, Periostin, *Cpxm2*, Inactive Carboxypeptidase-Like Protein X2. (**C**) (males) and (**D**) (females). Downregulated genes and genes have different modulations with aging between Gx and non-Gx animals or between males and females. *Slc38a1*, Solute Carrier Family 38 Member 1, *CD74*, CD molecule, *Fmo2*, Flavin Containing Dimethylaniline Monooxygenase 2 and *Zbtb16*, Zinc Finger and BTB Domain Containing 16. Results are expressed as the mean ± SEM (*n* = 4). One-way ANOVA followed by Holm-Sidak post-test. ^*^p < 0.05, ^**^p < 0.01, ^***^p < 0.001 and ^****^p < 0.0001 between indicated groups.

Myosin high chain beta (*Myh7*), Myotilin (*Myot*), 3-Hydroxybutyrate Dehydrogenase 1 (*Bdh1*), periostin (*Postn*) and the carboxypeptidase x, m14 family member 2 (*Cpxm2*) were five up-regulated genes shared between male and female mice having the highest levels of expression in the LV. (Figure 7A, 7B). *Slc38a1* (Solute Carrier Family 38 Member 1) was the most abundant down-regulated mRNA at 24 months in both sexes (Figures 5A, 5B and 7C, 7D).

Several highly expressed genes had levels not only modulated by age but also by the loss of sex steroids in elderly mice, such as CD74, which was more modulated in Gx animals, and Fmo2 and Zbtb16, showing the opposite trend (Figure 7C, 7D).

## DISCUSSION

In this study, we investigated the relative contribution of biological sex, loss of gonadal steroids, and sex chromosomes to heart morphology and function in mice at different times of life. Our objective was to understand better these factors’ contribution to the future development of heart disease models in aging mice.

We show that cardiac hypertrophy is an ongoing process in female mice, whereas, in males, this process slows and stops during the second year of life. In females, gonadal steroids continue to drive cardiac hypertrophy in aging mice, and the removal of ovaries mostly stopped this. The loss of gonadal steroids later in life in males also resulted in smaller hearts one year later at 24 months. Indexed heart weight was smaller at 24 months for Gx males than at the time of the gonadectomy at 12 months. This suggests that male gonadal hormones are essential for the heart to maintain its size later in life.

In young castrated (Gx) males, cardiac growth was reduced, showing the importance of male hormones’ importance. In young Gx females, this influence of female hormones on cardiac growth was not observed as previously described [27]. Loss of male sex steroids in mice is less clinically relevant, although testosterone levels are known to become lower in aging men [32]. Low testosterone levels are associated with cardiovascular risk and diseases, adverse cardiac events, and mortality due to those events [33]. In our mice, losing androgens later in life also stopped heart weight gain with age.

We allowed our mice to have an “active” lifestyle by enabling them to exercise voluntarily. A recent study studying cardiac aging in non-Gx mice of both sexes reported that signs of cardiac aging were evident at 18 months, especially in females, with an important reduction of LV volumes [34]. This was accompanied by changes in echo LV strain parameters at 18 months. This suggests that our mice may have kept their normal cardiac health longer by remaining active. The LV remodelled with age, but diastolic function remained mostly unchanged.

Ovariectomy of female mice has often been used as a surrogate for the study of menopause in preclinical models [35]. Perhaps surprisingly, the effects of Gx later in life have received very little attention in the past, although they may be more relevant to the human situation [36, 37]. As mentioned above, we observed that loss of ovarian steroids in 12-month-old females led to a stoppage of the ongoing age-related cardiac hypertrophy and less concentric LV remodelling. Twelve-month-old mice are believed to correspond to humans in their forties [38]. Ovariectomy of 12-month-old female mice did not result in increased body weight gain, unlike in younger animals, which became obese. The increased running activity in older females likely countered the obesity resulting from Gx in young mice. On the other hand, at 12 months, Gx caused a gain in body weight up to 18 months, remaining stable for the last six months (Supplementary Figure 1). We did not monitor the running activity of our mice, so we do not know if Gx mice had a different running behaviour than control females.

We observed that cardiac hypertrophy and remodelling in mice is an ongoing process influenced by age, biological sex, and sex steroid hormones. In humans, the same factors influence cardiac remodelling and hypertrophy [39]. Over a lifetime, left ventricular (LV) mass increases with age in women with a low burden of risk factors and decreases slightly in men. However, structural evaluations suggest more significant LV mass and wall thickness in men than women [40]. Our observations in female mice show cardiac hypertrophy, even in older animals, strongly depends on hormonal status. However, the result is like that of older women, i.e., cardiac hypertrophy, LV concentric remodelling, increased myocardial fibrosis, and cardiomyocytes CSA. Gonadectomy resulted in a somewhat less severe LV concentric phenotype.

Menopause, as understood for women, does not happen in most mammals. In mice, ovaries do not fail with age as in women, but the uterine capacity to sustain pregnancies declines with time [41]. The outcome is similar for reproduction capacity. The impact of ovarian steroids can continue in mice later in life, and our results suggest that their contribution must be considered since they influence cardiac morphology. The decrease in sex steroids in a Gx mouse model is drastic compared to being more gradual in menopausal women. We tried to circumvent this by studying animals many months after the loss of sex steroids. We cannot exclude that early events after the gonadectomy *per se* or during the peri-menopausal stage in women may have lasting effects on cardiac aging. It will be interesting to study a different model of menopause in mice to see if this reflects differences in cardiac morphology and function months later [23]. A recent study investigated the vinylcyclohexene dioxide (VCD) menopause model used to accelerate ovarian failure in young female mice. Menopause in this murine model was associated with a higher density of infiltrating cardiac immune cells and alterations of echo LV strain parameters [24].

Our results from FCG mice show that sex chromosomes play a limited role, if any, in cardiac growth and hypertrophy linked to aging. To study the contribution of sex chromosomes on a phenotype, removing the powerful effects of sex steroids is obligatory. Here, we investigated the four-core genotype mice at two life stages: adult mice at six months and elderly mice at 20 months. Doubling X chromosome dosage was associated with increased body weight and growth, but this was not the case for the heart. Echocardiography data did not show marked changes related to the number of X chromosomes except for LV wall thickness in older females, which was thinner in XY females. This is a potentially important observation that sex differences observed in heart disease phenotypes in elderly patients are probably more related to the effects of sex steroids earlier in life than on sex chromosomes.

Gonadectomy was not performed at the same age as for C57Bl6/J wild-type mice we also studied. Six-month-old mice had their gonads removed at the age of 5 weeks, whereas elderly mice had theirs removed at the age of 6 months. The follow-up of older FCG mice was thus 14 months instead of 12 months for wild-type animals. Interestingly, some sex chromosome effects were still apparent in non-Gx mice, namely LV walls being thinner in XY females compared to XX mice, suggesting that a possible gene escaping X inactivation or a gene on the Y chromosome may be implicated in the heart morphology in aging mice. We observed that four Y chromosome genes had relatively high levels of expression in the LV: Ddx3y, Eif2s3y, Uty, and Kdm5d (Supplementary Table 6). The roles of these genes in somatic cells are still not understood but could play a role considering their expression in the myocardium [42].

Unfortunately, our RNA-Seq data did not reveal many genes differentially expressed between older control and Gx females. This may be linked to the fact that by analyzing the LV transcriptome one year after Gx, we may have missed many earlier gene expression adaptations after losing gonadal steroids. We observed the same in males after castration at 12 months (only ten genes were differentially expressed in old Gx males and intact males). Gx increased body weight and tibial length growth and reduced cardiac hypertrophy compared to control animals. Still, only a few LV genes were expressed differently at 24 months between Gx and non-Gx animals.

Using RNA sequencing, we identified new genes associated with myocardial aging, namely *Bdh1*, *Myot, Cpxm2* and *Slc38a1*. These genes were modulated in both male and female mice but not by Gx.

The *Bdh1* gene encodes for the 3-Hydroxybutyrate Dehydrogenase 1 enzyme, which is responsible for the interconversion of acetoacetate and 3-hydroxybutyrate, the two major ketone bodies produced during fatty acid catabolism [43]. This may indicate that the aging heart may rely more on the oxidation of ketone bodies as an energy substrate, but this remains to be confirmed [44].

The *Myot* gene encodes for Myotilin, a thin filament-associated protein localized at the Z-disk of skeletal and cardiac muscle cells [45]. Myotilin is believed to be involved in the structural maintenance and function of the sarcomere. Loss of myotilin in mice does not result in a shorter lifespan or anomalies in skeletal and cardiac structure and function. Its expression is more substantial in skeletal muscles, and myotilin variants are known to cause severe forms of myopathies in humans [46]. Missense variants in the MYOT human gene can cause myopathy by accumulating protein aggregates [47]. The MYOT variant has been described in a case of cardiomyopathy with low ejection fraction; other patients displayed left bundle branch block or congestive heart failure [46]. Here, we do not know if higher Myot gene expression will result in older mice in the MYOT accumulation in cardiomyocyte Z-disks, and this could be an interesting hypothesis to test.

*Cpxm2* gene encodes the Inactive Carboxypeptidase-Like Protein X2. The role of CPXM2 is still obscure, but recent publications have associated it with cardiac hypertrophy and failure [48, 49]. CPXM2 protein was shown to be expressed along the t-tubule network and to colocalize with dihydropyridine receptors in cardiac myocytes. It is also expressed in cardiac fibroblasts. Its expression was increased in various models of cardiac hypertrophy in mice and rats and endocardial biopsies of patients with CH [48]. Again, higher Cpxm2 expression in aging hearts could be linked to the cardiac phenotype observed, but this remains to be studied.

SLC38A1 (Solute Carrier Family 38 Member 1) is an amino acid transporter and the primary glutamine transporter. Its expression was reduced with age in the hearts of our mice. A similar observation has been made in human skeletal muscle with age [50]. Since SLC38A1 is implicated in the cell’s uptake of short-chain neutral amino acids, this could impair the normal physiology of cardiac myocytes of older animals. In the heart, the role of this protein has yet to be studied.

## CONCLUSION

This study emphasizes that cardiac aging in mice depends on the hormonal status and the age at which this status is modified. In female mice, the heart gains mass during the first two years of life, and this cardiac hypertrophy is, at least in part, controlled by the presence of ovarian hormones during the second year. In males, the absence of testicular hormones reduces cardiac growth in young and adult animals. We observed a trend for cardiac atrophy in older animals.

Again, we feel that those considerations related to sex steroids should be considered when older mice are used to study cardiac physiology and diseases.

## Supplementary Materials

Supplementary Figure 1

Supplementary Tables

## References

[1] Ji H, Kwan AC, Chen MT, Ouyang D, Ebinger JE, Bell SP, Niiranen TJ, Bello NA, Cheng S. Sex Differences in Myocardial and Vascular Aging. Circ Res. 2022; 130:566–77. 10.1161/CIRCRESAHA.121.319902 35175845 PMC8863105

[2] Ponikowski P, Voors AA, Anker SD, Bueno H, Cleland JGF, Coats AJS, Falk V, González-Juanatey JR, Harjola VP, Jankowska EA, Jessup M, Linde C, Nihoyannopoulos P, et al. 2016 ESC Guidelines for the Diagnosis and Treatment of Acute and Chronic Heart Failure. Rev Esp Cardiol (Engl Ed). 2016; 69:1167. 10.1016/j.rec.2016.11.005 27894487

[3] Schiattarella GG, Altamirano F, Tong D, French KM, Villalobos E, Kim SY, Luo X, Jiang N, May HI, Wang ZV, Hill TM, Mammen PPA, Huang J, et al. Nitrosative stress drives heart failure with preserved ejection fraction. Nature. 2019; 568:351–6. 10.1038/s41586-019-1100-z 30971818 PMC6635957

[4] Withaar C, Meems LMG, Markousis-Mavrogenis G, Boogerd CJ, Silljé HHW, Schouten EM, Dokter MM, Voors AA, Westenbrink BD, Lam CSP, de Boer RA. The effects of liraglutide and dapagliflozin on cardiac function and structure in a multi-hit mouse model of heart failure with preserved ejection fraction. Cardiovasc Res. 2021; 117:2108–24. 10.1093/cvr/cvaa256 32871009 PMC8318109

[5] Tong D, Schiattarella GG, Jiang N, May HI, Lavandero S, Gillette TG, Hill JA. Female Sex Is Protective in a Preclinical Model of Heart Failure With Preserved Ejection Fraction. Circulation. 2019; 140:1769–71. 10.1161/CIRCULATIONAHA.119.042267 31738599 PMC6993895

[6] Withaar C, Lam CSP, Schiattarella GG, de Boer RA, Meems LMG. Heart failure with preserved ejection fraction in humans and mice: embracing clinical complexity in mouse models. Eur Heart J. 2021; 42:4420–30. 10.1093/eurheartj/ehab389 34414416 PMC8599003

[7] Shah SJ, Kitzman DW, Borlaug BA, van Heerebeek L, Zile MR, Kass DA, Paulus WJ. Phenotype-Specific Treatment of Heart Failure With Preserved Ejection Fraction: A Multiorgan Roadmap. Circulation. 2016; 134:73–90. 10.1161/CIRCULATIONAHA.116.021884 27358439 PMC4930115

[8] Savarese G, Lund LH. Global Public Health Burden of Heart Failure. Card Fail Rev. 2017; 3:7–11. 10.15420/cfr.2016:25:2 28785469 PMC5494150

[9] Koch SE, Haworth KJ, Robbins N, Smith MA, Lather N, Anjak A, Jiang M, Varma P, Jones WK, Rubinstein J. Age- and gender-related changes in ventricular performance in wild-type FVB/N mice as evaluated by conventional and vector velocity echocardiography imaging: a retrospective study. Ultrasound Med Biol. 2013; 39:2034–43. 10.1016/j.ultrasmedbio.2013.04.002 23791351 PMC4857602

[10] Zhang TY, Zhao BJ, Wang T, Wang J. Effect of aging and sex on cardiovascular structure and function in wildtype mice assessed with echocardiography. Sci Rep. 2021; 11:22800. 10.1038/s41598-021-02196-0 34815485 PMC8611093

[11] Kararigas G, Dworatzek E, Petrov G, Summer H, Schulze TM, Baczko I, Knosalla C, Golz S, Hetzer R, Regitz-Zagrosek V. Sex-dependent regulation of fibrosis and inflammation in human left ventricular remodelling under pressure overload. Eur J Heart Fail. 2014; 16:1160–7. 10.1002/ejhf.171 25287281

[12] Gaignebet L, Kańduła MM, Lehmann D, Knosalla C, Kreil DP, Kararigas G. Sex-Specific Human Cardiomyocyte Gene Regulation in Left Ventricular Pressure Overload. Mayo Clin Proc. 2020; 95:688–97. 10.1016/j.mayocp.2019.11.026 31954524

[13] Regitz-Zagrosek V, Kararigas G. Mechanistic Pathways of Sex Differences in Cardiovascular Disease. Physiol Rev. 2017; 97:1–37. 10.1152/physrev.00021.2015 27807199

[14] Lim WK, Wren B, Jepson N, Roy S, Caplan G. Effect of hormone replacement therapy on left ventricular hypertrophy. Am J Cardiol. 1999; 83:1132–4. 10.1016/s0002-9149(99)00029-6 10190535

[15] Light KC, Hinderliter AL, West SG, Grewen KM, Steege JF, Sherwood A, Girdler SS. Hormone replacement improves hemodynamic profile and left ventricular geometry in hypertensive and normotensive postmenopausal women. J Hypertens. 2001; 19:269–78. 10.1097/00004872-200102000-00014 11212970

[16] Mori T, Kai H, Kajimoto H, Koga M, Kudo H, Takayama N, Yasuoka S, Anegawa T, Kai M, Imaizumi T. Enhanced cardiac inflammation and fibrosis in ovariectomized hypertensive rats: a possible mechanism of diastolic dysfunction in postmenopausal women. Hypertens Res. 2011; 34:496–502. 10.1038/hr.2010.261 21248760

[17] Maric-Bilkan C, Arnold AP, Taylor DA, Dwinell M, Howlett SE, Wenger N, Reckelhoff JF, Sandberg K, Churchill G, Levin E, Lundberg MS. Report of the National Heart, Lung, and Blood Institute Working Group on Sex Differences Research in Cardiovascular Disease: Scientific Questions and Challenges. Hypertension. 2016; 67:802–7. 10.1161/HYPERTENSIONAHA.115.06967 26975706 PMC4833564

[18] Burgoyne PS, Arnold AP. A primer on the use of mouse models for identifying direct sex chromosome effects that cause sex differences in non-gonadal tissues. Biol Sex Differ. 2016; 7:68. 10.1186/s13293-016-0115-5 27999654 PMC5154145

[19] Li J, Chen X, McClusky R, Ruiz-Sundstrom M, Itoh Y, Umar S, Arnold AP, Eghbali M. The number of X chromosomes influences protection from cardiac ischaemia/reperfusion injury in mice: one X is better than two. Cardiovasc Res. 2014; 102:375–84. 10.1093/cvr/cvu064 24654234 PMC4030514

[20] Chen X, McClusky R, Itoh Y, Reue K, Arnold AP. X and Y chromosome complement influence adiposity and metabolism in mice. Endocrinology. 2013; 154:1092–104. 10.1210/en.2012-2098 23397033 PMC3578992

[21] Link JC, Chen X, Prien C, Borja MS, Hammerson B, Oda MN, Arnold AP, Reue K. Increased high-density lipoprotein cholesterol levels in mice with XX versus XY sex chromosomes. Arterioscler Thromb Vasc Biol. 2015; 35:1778–86. 10.1161/ATVBAHA.115.305460 26112012 PMC4668127

[22] Arnold AP, Cassis LA, Eghbali M, Reue K, Sandberg K. Sex Hormones and Sex Chromosomes Cause Sex Differences in the Development of Cardiovascular Diseases. Arterioscler Thromb Vasc Biol. 2017; 37:746–56. 10.1161/ATVBAHA.116.307301 28279969 PMC5437981

[23] Aidara ML, Walsh-Wilkinson É, Thibodeau SÈ, Labbé EA, Morin-Grandmont A, Gagnon G, Boudreau DK, Arsenault M, Bossé Y, Couët J. Cardiac reverse remodeling in a mouse model with many phenotypical features of heart failure with preserved ejection fraction: effects of modifying lifestyle. Am J Physiol Heart Circ Physiol. 2024; 326:H1017–36. 10.1152/ajpheart.00462.2023 38363584

[24] Van Kempen TA, Milner TA, Waters EM. Accelerated ovarian failure: a novel, chemically induced animal model of menopause. Brain Res. 2011; 1379:176–87. 10.1016/j.brainres.2010.12.064 21211517 PMC3078694

[25] Troy AM, Normukhamedova D, Grothe D, Momen A, Zhou YQ, McFadden M, Hussain M, Billia F, Cheng HM. Impact of ovary-intact menopause in a mouse model of heart failure with preserved ejection fraction. Am J Physiol Heart Circ Physiol. 2024; 326:H522–37. 10.1152/ajpheart.00733.2023 38180450 PMC11221814

[26] Arnold AP. Mouse models for evaluating sex chromosome effects that cause sex differences in non-gonadal tissues. J Neuroendocrinol. 2009; 21:377–86. 10.1111/j.1365-2826.2009.01831.x 19207816 PMC2669494

[27] Arnold AP, Chen X. What does the "four core genotypes" mouse model tell us about sex differences in the brain and other tissues? Front Neuroendocrinol. 2009; 30:1–9. 10.1016/j.yfrne.2008.11.001 19028515 PMC3282561

[28] Walsh-Wilkinson É, Aidara ML, Morin-Grandmont A, Thibodeau SÈ, Gagnon J, Genest M, Arsenault M, Couet J. Age and sex hormones modulate left ventricle regional response to angiotensin II in male and female mice. Am J Physiol Heart Circ Physiol. 2022; 323:H643–58. 10.1152/ajpheart.00044.2022 35984762

[29] Walsh-Wilkinson E, Arsenault M, Couet J. Segmental analysis by speckle-tracking echocardiography of the left ventricle response to isoproterenol in male and female mice. PeerJ. 2021; 9:e11085. 10.7717/peerj.11085 33763310 PMC7958899

[30] Ashburner M, Ball CA, Blake JA, Botstein D, Butler H, Cherry JM, Davis AP, Dolinski K, Dwight SS, Eppig JT, Harris MA, Hill DP, Issel-Tarver L, et al. Gene ontology: tool for the unification of biology. The Gene Ontology Consortium. Nat Genet. 2000; 25:25–9. 10.1038/75556 10802651 PMC3037419

[31] Aleksander SA, Balhoff J, Carbon S, Cherry JM, Drabkin HJ, Ebert D, Feuermann M, Gaudet P, Harris NL, Hill DP, Lee R, Mi H, Moxon S, et al, and Gene Ontology Consortium. The Gene Ontology knowledgebase in 2023. Genetics. 2023; 224:iyad031. 10.1093/genetics/iyad031 36866529 PMC10158837

[32] Feldman HA, Longcope C, Derby CA, Johannes CB, Araujo AB, Coviello AD, Bremner WJ, McKinlay JB. Age trends in the level of serum testosterone and other hormones in middle-aged men: longitudinal results from the Massachusetts male aging study. J Clin Endocrinol Metab. 2002; 87:589–98. 10.1210/jcem.87.2.8201 11836290

[33] Di Lodovico E, Facondo P, Delbarba A, Pezzaioli LC, Maffezzoni F, Cappelli C, Ferlin A. Testosterone, Hypogonadism, and Heart Failure. Circ Heart Fail. 2022; 15:e008755. 10.1161/CIRCHEARTFAILURE.121.008755 35392658

[34] Grilo GA, Shaver PR, Stoffel HJ, Morrow CA, Johnson OT, Iyer RP, de Castro Brás LE. Age- and sex-dependent differences in extracellular matrix metabolism associate with cardiac functional and structural changes. J Mol Cell Cardiol. 2020; 139:62–74. 10.1016/j.yjmcc.2020.01.005 31978395 PMC11017332

[35] Joll JE 2nd, Bersi MR, Nyman JS, Merryman WD. Evaluation of early bilateral ovariectomy in mice as a model of left heart disease. Am J Physiol Heart Circ Physiol. 2022; 322:H1080–5. 10.1152/ajpheart.00157.2022 35486477 PMC9142153

[36] Glassberg MK, Choi R, Manzoli V, Shahzeidi S, Rauschkolb P, Voswinckel R, Aliniazee M, Xia X, Elliot SJ. 17β-estradiol replacement reverses age-related lung disease in estrogen-deficient C57BL/6J mice. Endocrinology. 2014; 155:441–8. 10.1210/en.2013-1345 24274985 PMC3891937

[37] Tomicek NJ, Miller-Lee JL, Hunter JC, Korzick DH. Estrogen receptor beta does not influence ischemic tolerance in the aged female rat heart. Cardiovasc Ther. 2013; 31:32–7. 10.1111/j.1755-5922.2011.00288.x 21884022 PMC3235240

[38] Dutta S, Sengupta P. Men and mice: Relating their ages. Life Sci. 2016; 152:244–8. 10.1016/j.lfs.2015.10.025 26596563

[39] Kane AE, Howlett SE. Differences in Cardiovascular Aging in Men and Women. Adv Exp Med Biol. 2018; 1065:389–411. 10.1007/978-3-319-77932-4_25 30051398

[40] Lieb W, Xanthakis V, Sullivan LM, Aragam J, Pencina MJ, Larson MG, Benjamin EJ, Vasan RS. Longitudinal tracking of left ventricular mass over the adult life course: clinical correlates of short- and long-term change in the framingham offspring study. Circulation. 2009; 119:3085–92. 10.1161/CIRCULATIONAHA.108.824243 19506113 PMC2761217

[41] Finn CA. Reproductive ageing and the menopause. Int J Dev Biol. 2001; 45:613–7. 11417906

[42] Deschepper CF. Regulatory effects of the Uty/Ddx3y locus on neighboring chromosome Y genes and autosomal mRNA transcripts in adult mouse non-reproductive cells. Sci Rep. 2020; 10:14900. 10.1038/s41598-020-71447-3 32913328 PMC7484786

[43] Kadir AA, Stubbs BJ, Chong CR, Lee H, Cole M, Carr C, Hauton D, McCullagh J, Evans RD, Clarke K. On the interdependence of ketone body oxidation, glycogen content, glycolysis and energy metabolism in the heart. J Physiol. 2023; 601:1207–24. 10.1113/JP284270 36799478 PMC10684314

[44] Deng Y, Xie M, Li Q, Xu X, Ou W, Zhang Y, Xiao H, Yu H, Zheng Y, Liang Y, Jiang C, Chen G, Du D, et al. Targeting Mitochondria-Inflammation Circuit by β-Hydroxybutyrate Mitigates HFpEF. Circ Res. 2021; 128:232–45. 10.1161/CIRCRESAHA.120.317933 33176578

[45] Wadmore K, Azad AJ, Gehmlich K. The Role of Z-disc Proteins in Myopathy and Cardiomyopathy. Int J Mol Sci. 2021; 22:3058. 10.3390/ijms22063058 33802723 PMC8002584

[46] Moza M, Mologni L, Trokovic R, Faulkner G, Partanen J, Carpén O. Targeted deletion of the muscular dystrophy gene myotilin does not perturb muscle structure or function in mice. Mol Cell Biol. 2007; 27:244–52. 10.1128/MCB.00561-06 17074808 PMC1800670

[47] Selcen D, Engel AG. Mutations in myotilin cause myofibrillar myopathy. Neurology. 2004; 62:1363–71. 10.1212/01.wnl.0000123576.74801.75 15111675

[48] Grabowski K, Herlan L, Witten A, Qadri F, Eisenreich A, Lindner D, Schädlich M, Schulz A, Subrova J, Mhatre KN, Primessnig U, Plehm R, van Linthout S, et al. Cpxm2 as a novel candidate for cardiac hypertrophy and failure in hypertension. Hypertens Res. 2022; 45:292–307. 10.1038/s41440-021-00826-8 34916661 PMC8766285

[49] Subrova J, Böhme K, Gillespie A, Orphal M, Plum C, Kreutz R, Eisenreich A. MiRNA-29b and miRNA-497 Modulate the Expression of Carboxypeptidase X Member 2, a Candidate Gene Associated with Left Ventricular Hypertrophy. Int J Mol Sci. 2022; 23:2263. 10.3390/ijms23042263 35216380 PMC8880112

[50] Giresi PG, Stevenson EJ, Theilhaber J, Koncarevic A, Parkington J, Fielding RA, Kandarian SC. Identification of a molecular signature of sarcopenia. Physiol Genomics. 2005; 21:253–63. 10.1152/physiolgenomics.00249.2004 15687482

