# Supplementary material for: Biological sex, sex steroids and sex chromosomes contribute to mouse cardiac aging
Source: Aging (Albany NY). 2024 May 13;16(9):7553–77. doi: 10.18632/aging.205822 (PMC11131996; doi:10.18632/aging.205822)
Supplement: Supplementary Figure 1 [file aging-16-205822-s001.pdf]

## SUPPLEMENTARY FIGURE

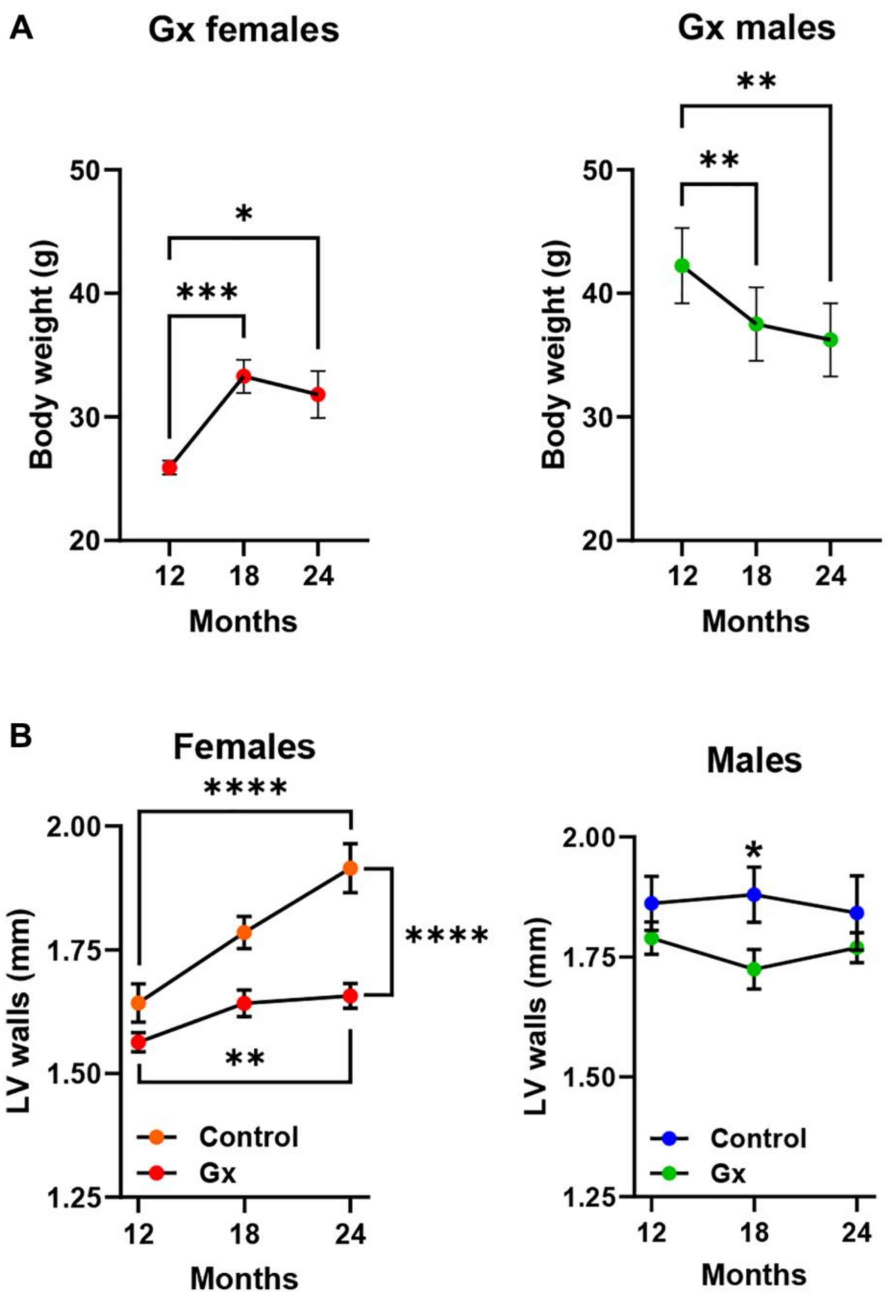


**Supplementary Figure 1.** Progression of body weight (**A**) and LV walls thickness (**B**) in older mice after gonadectomy compared to age-matched controls. Results are expressed as the mean ± SEM. (**A**, **B**) Statistical analysis by one-way ANOVA followed by Holm-Sidak post-test. $^{*}p < 0.05$, $^{**}p < 0.01$ and $^{****}p < 0.0001$ between indicated groups.
