# Supplementary material for: Biological sex, sex steroids and sex chromosomes contribute to mouse cardiac aging
Source: Aging (Albany NY). 2024 May 13;16(9):7553–77. doi: 10.18632/aging.205822 (PMC11131996; doi:10.18632/aging.205822)
Supplement: Supplementary Tables [file aging-16-205822-s002.pdf]

SUPPLEMENTARY TABLES

Supplementary Table 1. Means ± SEM of several morphological parameters for male and female mice at 3, 12 and 24 months, Gx or not.

**Males**	**3 m**	**12 m**	**24 m**
Controls			
Heart, mg	130 ± 4.0	167 ± 9.0	180 ± 6.5
Lungs, mg	128 ± 1.5	182 ± 8.2	189 ± 7.9
Left atrium, mg	3.6± 0.34	---	6.6 ± 0.41
Body weight, g	25.9 ± 0.62	36.7 ± 1.58	35 ± 1.6
Tibial length, mm	22.6 ± 0.06	22.6 ± 0.06	23.2 ± 0.15
Gx			
Heart, mg	---	139.3 ± 5.2	157 ± 4.4
Lungs, mg	---	158 ± 8.1	185 ± 5.9
Left atrium, mg	---	---	4.6 ± 0.26
Body weight, g	---	31.4 ± 1.95	37.6 ± 2.17
Tibial length, mm	---	23.4 ± 0.17	23.6 ± 0.14
**Females**	3 m	12 m	24 m
Controls			
Heart, mg	101 ± 2.0	122 ± 4.7	150 ± 4.6
Lungs, mg	118 ± 2.3	168 ± 5.9	174 ± 5.8
Left atrium, mg	2.8 ± 0.11	---	4.8 ± 0.43
Body weight, g	19.4 ± 0.29	28.5 ± 0.58	30.3 ± 1.76
Tibial length, mm	21.7 ± 0.10	22.1 ± 0.09	22.9 ± 0.15
Gx			
Heart, mg	---	139.3 ± 5.2	129 ± 3.4
Lungs, mg	---	158 ± 8.1	178 ± 5.9
Left atrium, mg	---	---	3.3 ± 0.21
Body weight, g	---	31.4 ± 1.95	31.8 ± 1.90
Tibial length, mm	---	23.4 ± 0.17	23.6 ± 0.14

Supplementary Table 2. Echocardiography (Echo) data from young FCG mice at 3 months.

**Parameter**	**MXY** ***N* = 13**	**FXX** ***N* = 14**	**MXX** ***N* = 15**	**FXY** ***N* = 20**
M-Mode				
EDD, mm	3,8 ± 0,05	3.7 ± 0,04	3,9 ± 0,04	3.7 ± 0,02
ESD, mm	2,7 ± 0,06	2.6 ± 0,06	2,7 ± 0,06	2.7 ± 0,05
PW, mm	0,70 ± 0,01	0.70 ± 0,01	0,75 ± 0,01*	0,69 ± 0,01
IVS, mm	0,70 ± 0,01	0,67 ± 0,01	0,72 ± 0,01	0,67 ± 0,01
RWT	0,34 ± 0,01	0,37 ± 0,01	0,38 ± 0,01*	0,37 ± 0,01
Simpson's				
SV, mm	31,3 ± 0,83	26.6 ± 0.66	34.2 ± 1,13	26.8 ± 0.72
EF, %	56,9 ± 1.7	57.5 ± 1.3	58.4 ± 1.4	55.8 ± 1.20
HR, bpm	444 ± 7.21	434 ± 7.8	447 ± 4.9	444 ± 5.33
CO, ml/min	13.9 ± 0,50	11.5 ± 0,32	15.3 ± 0,69	11.9 ± 0.32
EDV, µl	55.5 ± 2,07	45.5 ± 1.13	58.6 ± 1.42	48.0 ± 0.85

ESV, µl	24.2 ± 1.47	19.9 ± 0.92	24.5 ± 1.02	21.2 ± 0.69
Doppler				
E, mm/s	725 ± 25.5	663 ± 8.4	737 ± 8.53	714 ± 13.9
A, mm/s	402 ± 22.4	354 ± 12.1	461 ± 14.6	385 ± 11.5
E/A	1.85 ± 0,05	1.92 ± 0,07	1.64 ± 0,05*	1.88 ± 0,044
E', mm/s	−25.1 ± 0,99	−25.7 ± 1.07	−25.5 ± 0.56	−26.2 ± 0.71
A', mm/s	−17.9 ± 0,70	−17,6 ± 0,52	−19.2 ± 0,41	−18.8 ± 0.51
E/E'	−29.0 ± 0.98	−26.7 ± 1.19	−29.3 ± 0.75	−27.7 ± 0,76
E'/A'	1.41 ± 0,04	1.47 ± 0,04	1.34 ± 0,03	1.40 ± 0,03

Left ventricle parameters were measured in male and female mice, either XY or XX. EDD: end-diastolic LV diameter; ESD: end-systolic LV diameter; PW: diastolic posterior wall thickness; IVS: diastolic inter-ventricular septum thickness; RWT: relative wall thickness; SV: Stroke volume; EF: Ejection fraction; HR: Heart rate; CO: Cardiac output; EDV: end-diastolic volume; ESV: end-systolic volume; E: E wave; A: A wave; E': E' wave; A': A' wave. Results are expressed as the mean ± standard error of the mean (SEM) from the indicated number of animals. Two-way ANOVA analysis and Holm-Sidak post-test. $^{*}p < 0.05$ vs. same sex animals.

Supplementary Table 3. Echocardiography (Echo) data from aging non-Gx FCG mice at 20 months.

Parameter	MXY *N* = 11	FXX *N* = 11	MXX *N* = 13	FXY *N* = 11
M-Mode				
EDD, mm	3,7 ± 0,04	3,9 ± 0,09	3,8 ± 0,06	3.8 ± 0,09
ESD, mm	2,2 ± 0,06	2,5 ± 0,11	2,4 ± 0,09	2.3 ± 0,13
PW, mm	0,81 ± 0,01	0,80 ± 0,02	0,82 ± 0,02	0,84 ± 0,01
IVS, mm	0,74 ± 0,01	0,74 ± 0,02	0,75 ± 0,01	0,75 ± 0,02
RWT	0,42 ± 0,01	0,40 ± 0,01	0,42 ± 0,01	0,42 ± 0,01
Simpson's				
SV, mm	32.5 ± 1.19	34.5± 2.20	29.8 ± 0.87	32.6 ± 1.35
EF, %	63 ± 1,2	62.2 ± 1.84	61.2 ± 1.83	63.6 ± 1.7
HR, bpm	501 ± 12.7	492 ± 22.8	515 ± 12.3	488 ± 12.4
CO, ml/min	16.3 ± 0,69	16.7 ± 1.01	15,4 ± 0,55	16.0 ± 0.93
EDV, µl	51.3 ± 1,64	55.5 ± 3.49	49.4 ± 2,30	51.5 ± 2.4
ESV, µl	18.9 ± 0.88	21.02 ± 1.68	19.6 ± 1.79	21.1 ± 0.59
Doppler				
E, mm/s	660 ± 27.7	636 ± 28.4	569 ± 21.5*	683 ± 20.9
A, mm/s	434 ± 17.6	423 ± 17.1	379 ± 9.1*	437 ± 17.0
E/A	1,52 ± 0,02	1,52 ± 0,03	1.50 ± 0,05	1.61 ± 0,04
E', mm/s	−31.1 ± 1.33	−31.4 ± 0.99	−29.8 ± 0,59	−32.0 ± 0.59
A', mm/s	−20.4 ± 1.19	−20.6 ± 0.47	−18.7 ± 0.59	−19.8 ± 0.83
E/E'	−21.7 ± 1.46	−20.0 ± 0.83	−19.2 ± 0.78	−21.4 ± 0.59
E'/A'	1.54 ± 0.02	1.52 ± 0,03	1.6 ± 0,04	1.59 ± 0,04

Left ventricle parameters were measured in male and female mice, either XY or XX. Abbreviations: EDD: end-diastolic LV diameter; ESD: end-systolic LV diameter; PW: diastolic posterior wall thickness; IVS: diastolic inter-ventricular septum thickness; RWT: relative wall thickness; SV: Stroke volume; EF: Ejection fraction; HR: Heart rate; CO: Cardiac output; EDV: end-diastolic volume; ESV: end-systolic volume; E: E wave; A: A wave; E': E' wave; A': A' wave. Results are expressed as the mean ± standard error of the mean (SEM) from the indicated number of animals. Two-way ANOVA analysis and Holm-Sidak post-test. $^{*}p < 0.05$ vs. same sex animals.

Supplementary Table 4. Echocardiography (Echo) data from young gonadectomized FCG mice at 3 months.

Parameter	MXY *N* = 8	FXX *N* = 8	MXX *N* = 6	FXY *N* = 8
M-Mode				
EDD, mm	3,7 ± 0,05	3.7 ± 0,04	3,9 ± 0,02	3.9 ± 0,02
ESD, mm	2,7 ± 0,08	2,5 ± 0,07	2,8 ± 0,07	2,8 ± 0,07
PW, mm	0,69 ± 0,02	0.74 ± 0,02	0,65 ± 0,02	0,65 ± 0,02**
IVS, mm	0,71 ± 0,02	0.72 ± 0,01	0,63 ± 0,02*	0,63 ± 0,01**
RWT	0,38 ± 0,01	0,39 ± 0,01	0,33 ± 0,01*	0,33 ± 0,01*
Simpson's				
SV, mm	29.1 ± 1.42	33.0 ± 0.67	34.2 ± 1.24	29,0 ± 1.14
EF, %	57.8 ± 2,43	66.2 ± 1,21	57.8 ± 2.62	54,7 ± 1,94*
HR, bpm	451 ± 13.0	480 ± 11.4	468 ± 18.3	407 ± 16,2*
CO, ml/min	13.2 ± 0.85	15.8 ± 0,41	15.0 ± 0.33	11.9 ± 0.88
EDV, µl	50.7 ± 2,01	49.9 ± 0.64	57.8 ± 2.61	53,0 ± 1.09
ESV, µl	21.6 ± 1.79	16.9 ± 0.66	25.5 ± 1.45	24.0 ± 1.15*
Doppler				
E, mm/s	694 ± 26.8	673 ± 17.1	711 ± 23.6	676 ± 21.7
A, mm/s	432 ± 20.4	437 ± 17.6	443 ± 8.41	376 ± 14.0*
E/A	1.63 ± 0,07	1.56 ± 0,04	1.60 ± 0,03	1.82 ± 0.07*
E', mm/s	−24.5 ± 1.43	−26.8 ± 1.65	−25.4 ± 0.97	−24.6 ± 1.35
A', mm/s	−17.5 ± 0.90	−17,7 ± 0,59	−18.1 ± 0.86	−19.5 ± 0.97
E/E'	−29.4 ± 1.99	−25.9 ± 1.36	−28.9 ± 1.53	−27.9 ± 0,78
E'/A'	1,42 ± 0,08	1,51 ± 0,08	1.42 ± 0,08	1,26 ± 0,01**

Left ventricle parameters were measured in male and female mice, either XY or XX. Abbreviations: EDD: end-diastolic LV diameter; ESD: end-systolic LV diameter; PW: diastolic posterior wall thickness; IVS: diastolic inter-ventricular septum thickness; RWT: relative wall thickness; SV: Stroke volume; EF: Ejection fraction; HR: Heart rate; CO: Cardiac output; EDV: end-diastolic volume; ESV: end-systolic volume; E: E wave; A: A wave; E': E' wave; A': A' wave. Results are expressed as the mean ± standard error of the mean (SEM) from the indicated number of animals. Two-way ANOVA analysis and Holm-Sidak post-test. $^{*}p < 0.05$ and $^{**}p < 0.01$ vs. same sex animals.

Supplementary Table 5. Echocardiography (Echo) data from aging gonadectomized FCG mice at 20 months.

Parameter	MXY *N* = 7	FXX *N* = 5	MXX *N* = 7	FXY *N* = 7
M-Mode				
EDD, mm	3,7 ± 0,04	3,9 ± 0,10	3,8 ± 0,08	3.7 ± 0,09
ESD, mm	2,1 ± 0,07	2,3 ± 0,15	2,3 ± 0,10	2.0 ± 0,04
PW, mm	0,79 ± 0,02	0,86 ± 0,04	0,82 ± 0,02	0,84 ± 0,03
IVS, mm	0,70 ± 0,01	0,78 ± 0,03	0,76 ± 0,03	0,75 ± 0,02
RWT	0,40 ± 0,01	0,43 ± 0,02	0,42 ± 0,01	0,43 ± 0,01
Simpson's				
SV, mm	32.4 ± 1.41	35.5 ± 1.82	32.5 ± 1.69	32.9 ± 1.42
EF, %	65.4 ± 1.12	64.2 ± 2.44	64.2 ± 0.97	67.6 ± 1.0
HR, bpm	487 ± 16.3	508 ± 12.8	494 ± 7.2	497 ± 8.3
CO, ml/min	15.8 ± 0.84	18.0 ± 0.78	16.0 ± 0,89	16.3 ± 0.53
EDV, µl	49.5 ± 1.85	55.5 ± 3.59	50.6 ± 2.65	48.8 ± 2.2

ESV, µl	17.1 ± 0.81	20.0 ± 2.49	18.1 ± 1.13	15.9 ± 1.01
Doppler				
E, mm/s	607 ± 22.5	663 ± 26.5	632 ± 29.3	636 ± 31.2
A, mm/s	363 ± 9.6	390 ± 9.6	409 ± 10.9*	395 ± 14.0
E/A	1.70 ± 0,04	1.70 ± 0.06	1.55 ± 0,06	1.61 ± 0.03
E', mm/s	−29.6 ± 0.38	−29.6 ± 0.56	−30.8 ± 1.44	−28.5 ± 0.63
A', mm/s	−16.7 ± 0.39	−17.4 ± 1.15	−18.5 ± 1.26	−17.6 ± 0.70
E/E'	−20.5 ± 0.78	−22.4 ± 1.09	−20.6 ± 00.85	−22.3 ± 0.92
E'/A'	1.80 ± 0.04	1.74 ± 0.11	1.68 ± 0,06	1.63 ± 0,06

Left ventricle parameters were measured in male and female mice, either XY or XX. Abbreviations: EDD: end-diastolic LV diameter; ESD: end-systolic LV diameter; PW: diastolic posterior wall thickness; IVS: diastolic inter-ventricular septum thickness; RWT: relative wall thickness; SV: Stroke volume; EF: Ejection fraction; HR: Heart rate; CO: Cardiac output; EDV: end-diastolic volume; ESV: end-systolic volume; E: E wave; A: A wave; E': E' wave; A': A' wave. Results are expressed as the mean ± standard error of the mean (SEM) from the indicated number of animals. Two-way ANOVA analysis and Holm-Sidak post-test. $^{*}p < 0.05$ vs. same-sex animals.

Supplementary Table 6. Differentially regulated LV genes between 24-month-old males and 24-month-old females.

**Lower expression in females**			
**Gene**	**Mean count**	**log2(FC)**	***q*-Value**
**Ddx3y**	**775.2**	**−7.25**	**2.53E-04**
**Eif2s3y**	**526.89**	**−6.62**	**9.05E-04**
**Uty**	**227.66**	**−6.54**	**2.60E-05**
**Kdm5d**	**216.57**	**−6.23**	**0.00E+00**
Cyp26b1	953.58	−3.17	2.60E-05
C7	357.49	−2.45	0.00E+00
Ppbp	60.56	−2.24	1.46E-03
Itga2b	70.33	−1.79	2.10E-02
Atp2b2	51.79	−1.37	3.21E-03
Npas2	85.14	−1.3	3.08E-02
Kcnk1	113.37	−1.28	1.80E-04
Prom2	91.29	−1.18	8.38E-04
Tmem171	59.41	−1.17	4.95E-02
Aqp8	143.92	−1.1	3.52E-03
**Higher expression in females**			
**Xist**	**3969.72**	**9.24**	**0.00E+00**
Car3	52.27	2.64	7.00E-06
Lypd8l	129.99	1.47	6.12E-04
Col14a1	634.85	1.41	0.00E+00
Ptn	91.13	1.07	2.78E-02
Erdr1	1099.73	1.06	1.00E-06
Dkk3	229.81	1.06	7.85E-03
Kcne1	74.77	1.06	1.40E-02
Lepr	151.48	1.01	1.60E-03

Mean count: Normalized mean count (male vs. female), FC: fold change and *q*-value: the proportion of false positives among all positive results. In bold, sex chromosome genes.

Supplementary Table 7. Differentially regulated LV genes between 24-month-old males and 24-month-old Gx males.

Lower expression in Gx males			
**Gene**	**Mean Count**	**log2(FC)**	***q*-Value**
C7	364.22	−1.96	1.00E-06
Lcn2	60.24	−1.94	5.00E-06
Cnmd	70.31	−1.43	6.98E-03
Klk1b26	51.86	−1.32	3.66E-02
Cyp2b10	209.23	−1.28	2.26E-04
Mmp3	183.84	−1.04	6.38E-03
**Higher expression in Gx males**			
Lrrc55	56.78	2.15	0.00E+00
Col14a1	635.06	1.49	0.00E+00
Ptn	94.82	1.24	6.98E-03
Mdk	60.65	1.1	9.50E-03

Mean count: Normalized mean count (male vs. female), FC: fold change and *q*-value: the proportion of false positives among all positive results.

Supplementary Table 8. Differentially regulated LV genes between 24-month-old females and 24-month-old Gx females.

Lower expression in Gx females			
**Gene**	**Mean count**	**log2(FC)**	***q*-Value**
Fkbp5	672.41	−1.1	2.30E-05
Ifi205	379.99	−1.02	3.60E-05
Zbtb16	3608.46	−1	7.86E-03

Mean count: Normalized mean count (male vs. female), FC: fold change and *q*-value: the proportion of false positives among all positive results.
